# Comparing resting state fMRI de-noising approaches using multi- and single-echo acquisitions

**DOI:** 10.1371/journal.pone.0173289

**Published:** 2017-03-21

**Authors:** Ottavia Dipasquale, Arjun Sethi, Maria Marcella Laganà, Francesca Baglio, Giuseppe Baselli, Prantik Kundu, Neil A. Harrison, Mara Cercignani

**Affiliations:** 1 Department of Electronics, Information and Bioengineering, Politecnico di Milano, Milan, Italy; 2 Fondazione don Carlo Gnocchi ONLUS, IRCCS Santa Maria Nascente, Milan, Italy; 3 Clinical Imaging Sciences Centre, Brighton and Sussex Medical School, Brighton, United Kingdom; 4 Section on Advanced Functional Neuroimaging, Brain Imaging Center, Icahn School of Medicine at Mount Sinai, New York, United States of America; 5 Sussex Partnership NHS Foundation Trust, Brighton, United Kingdom; 6 Sackler Centre for Consciousness Science, University of Sussex, Brighton, United Kingdom; Istituto Italiano di Tecnologia, ITALY

## Abstract

Artifact removal in resting state fMRI (rfMRI) data remains a serious challenge, with even subtle head motion undermining reliability and reproducibility. Here we compared some of the most popular single-echo de-noising methods—regression of Motion parameters, White matter and Cerebrospinal fluid signals (MWC method), FMRIB’s ICA-based X-noiseifier (FIX) and ICA-based Automatic Removal Of Motion Artifacts (ICA-AROMA)—with a multi-echo approach (ME-ICA) that exploits the linear dependency of BOLD on the echo time. Data were acquired using a clinical scanner and included 30 young, healthy participants (minimal head motion) and 30 Attention Deficit Hyperactivity Disorder patients (greater head motion). De-noising effectiveness was assessed in terms of data quality after each cleanup procedure, ability to uncouple BOLD signal and motion and preservation of default mode network (DMN) functional connectivity. Most cleaning methods showed a positive impact on data quality. However, based on the investigated metrics, ME-ICA was the most robust. It minimized the impact of motion on FC even for high motion participants and preserved DMN functional connectivity structure. The high-quality results obtained using ME-ICA suggest that using a multi-echo EPI sequence, reliable rfMRI data can be obtained in a clinical setting.

## Introduction

Resting state functional MRI (rfMRI) is a powerful method for investigating functional connectivity (FC) in the healthy brain and neuropsychiatric disorders. However, artifact removal remains a serious and important challenge. This issue is intrinsic to rfMRI data analyses as images are acquired without an experimental modulation of brain function, thus no a-priori knowledge about the signal of interest. Consequently, discrimination of neural BOLD activity and a variety of spurious non-BOLD signals is more challenging than for task-based fMRI. Head motion is one of the most difficult artifacts due to its unpredictable and non-linear nature[1–5]. As demonstrated by recent studies, even subtle head motion (<0.5 mm) can seriously undermine reliability of results, tending to increase short-range and simultaneously decrease long-range connections[4–6]. This problem is particularly marked in clinical studies involving patients with neuropsychiatric disorders such as autism, Attention Deficit Hyperactivity Disorder (ADHD) and Tourette syndrome, neurodegenerative diseases such as Alzheimer’s disease and studies in young children where movement is often pronounced[7].

Several motion artifact removal methods have been proposed in the literature. The simplest consists of using motion parameters estimated during the realignment step of the rfMRI volumes as confounding regressors[5, 8, 9]. Typically, white matter (WM) and cerebrospinal fluid (CSF) mean signals are also included in these regression models, as BOLD signal related to neural activity should predominate in grey matter, while global drifts should affect all tissues[10]. Some studies also suggest using a complimentary strategy named “scrubbing” in order to exclude data volumes (time points) affected by excessive motion[2]. However, these strategies present some limitations. For example, these methods substantially overlook non-linear dynamics, while scrubbing can introduce biases due to the high and sometimes variable loss in temporal degrees of freedom[5, 7, 9, 11, 12].

An alternative approach is based on a multivariate data decomposition using independent component analysis (ICA)[13–18] followed by regression of the artifactual components. FMRIB’s ICA-based X-noiseifier (FIX)[18, 19] is based on this approach and uses an ICA component classifier for the automatic classification of good (BOLD signal) and bad (artifact) components. However, the effectiveness of this procedure relies on the existence of a reference set of good and bad components, which must be trained by hand. In fact, if the algorithm is not adequately trained, it can lead to suboptimal cleaning and bias subsequent data analyses[7]. More recently, an alternative ICA-based method called ICA-based Automatic Removal Of Motion Artifacts (ICA-AROMA) has been proposed[11]. This method uses an automatic classifier that categorizes each component as either BOLD signal or artifact based on its high-frequency content, and correlation with realignment parameters, edge and CSF fractions.

Another novel ICA-based cleaning approach has also been recently introduced[20]. The major difference between this and other ICA-based methods is that it utilizes a combination of a multi-echo (ME) rfMRI acquisition[21] and ICA analysis. This method, called ME-ICA, takes advantage of the distinctive characteristic of the BOLD T2* signal, whose percent signal change is linearly dependent on echo time (TE). Component-level TE dependence is measured using two pseudo-F-statistics, which evaluate BOLD and non-BOLD component weights by fitting the signal changes across TEs with two alternative models: one TE-dependent and one TE-independent. The resulting summary scores clearly differentiate Resting State Network (RSN) from non-BOLD-like components [16]. Finally, time courses assessed as non-BOLD signals are used as noise regressors for data cleaning. The main disadvantage of this method relies in its ineffectiveness with those BOLD-like components that cannot be removed completely due to their dependence on TE. Examples include T2* fluctuations in the WM and in sagittal and transverse draining veins.

This study aimed to compare the ability to minimize the impact of head motion in rfMRI analyses of the most popular single-echo methods, i.e. the regression of 24 motion parameters and WM and CSF signals (MWC method), FIX and ICA-AROMA, and the multi-echo approach (ME-ICA). Our analysis focused mainly on ICA-based methods, as their efficiency in removing unwanted motion-induced signals has been reliably demonstrated[7, 19, 22]. We also wanted to show the ability of ICA to substantially improve rfMRI data quality and preserve signal of interest. Methods based on regression of data obtained from external physiological recordings[23] were not included in the comparison, as physiological data were not available for this study. Scrubbing was not included either, as it has previously been shown that it can have a significant impact on the temporal autocorrelation structure of fMRI data and can lead to a high and variable loss in temporal degrees of freedom[5, 7, 9, 11, 12]. Moreover, it has already been demonstrated to be sub-optimal in removing the degrees of freedom from rfMRI data compared to most of the other methods examined here[7].

We compared the performance of the different cleaning approaches on data from young healthy controls (HC)—a typical low-motion group of subjects—and patients with ADHD—a population characterized by restlessness and a high degree of head movement—in order to evaluate the effectiveness of the artifact removal for both high and low movement groups. We first estimated data quality using established indices[2, 7, 19, 20, 22, 24, 25], such as Delta VARiation Signal (DVARS), temporal signal to noise ratio (SNR), power spectral density and number of temporal degrees of freedom lost after cleanup. We then evaluated the ability of each method to uncouple FC and motion, reduce distance-dependent connectivity biases[4–6] and preserve BOLD signal.

## Methods

### Participants and MRI data acquisition

Thirty patients with ADHD (34 ± 9.5 years, M/F: 19/11) were recruited from specialist clinics at Sussex Partnership NHS Foundation Trust (SPFT). All patients fulfilled DSM-IV criteria for ADHD. Patients with co-existing neurological or psychiatric co-morbidities including psychotic disorders, as well as concurrent anxiety or unipolar depressive disorder not currently in remission were excluded. Thirty age, sex, and IQ matched healthy controls (HC, 33 ± 9.5 years, M/F: 19/11) participated in the study and were recruited using on-line classified advertising websites and university mailing lists. Ethical approvals were obtained from the East of England: Hertfordshire National Research Ethics Committee (NRES) and the local BSMS Research Governance and Ethics Committee. All participants provided written informed consent.

Data were acquired using a 1.5 T Siemens Avanto MRI scanner (Siemens AG Medical Solutions, Erlangen, Germany) equipped with a 32-channel head-coil. Functional MRI data were obtained during rest using a T2*-weighted multi-echo EPI sequence[21] (TR = 2570 ms; TE = 15, 34, 54 ms; flip angle = 90°; resolution = 3.7 × 3.75 × 4.49 mm; matrix size = 64 x 64; 31 axial slices; 200 volumes). Guidelines for setting up the multi-echo acquisition are included in S1 Appendix.

A 3D T1-weighted anatomical scan was obtained for each participant in one session using an MP-RAGE acquisition (TR = 2730 ms, TE = 3.57 ms, TI = 1000 ms, flip angle = 7°, matrix = 256 x 240, number of partitions = 192, GRAPPA factor = 2, resolution = 1 mm^3^). This dataset is part of a larger study on ADHD, for which a number of other MRI sequences were collected from every participant (data not reported here).

### Image pre-processing

Two different types of image pre-processing were completed.

Multi-echo data pre-processing was performed using the AFNI[26] tool *meica*.*py*[20, 27]. Pre-processing steps included volume re-alignment, time-series de-spiking and slice time correction. Functional data were then optimally combined (OC) by taking a weighted summation of the three echoes, using an exponential T2* weighting approach[28].

The raw images obtained with TE = 54ms were separately analyzed as a single-echo acquisition and pre-processed with FSL[29, 30]. This TE was chosen as it is closest to the optimal TE for fMRI at 1.5T[31]. Standard pre-processing steps involved: volume re-alignment with MCFLIRT[32], non-brain tissue removal with the brain extraction tool (BET)[33] and spatial smoothing with a 5 mm full width at half maximum Gaussian kernel. Unlike the multi echo dataset, the single-echo dataset was not slice-timing corrected, as suggested by the FSL developers. This choice is justified by our principle of following the recommended pipeline for each method. However, in order to test whether slice-timing correction would significantly impact the performance of motion correction algorithms that can be plugged-in with FSL pre-processing, we also preprocessed single-echo data with additional slice timing correction and used ICA-AROMA to de-noise that dataset.

The six rigid-body parameters extracted for each participant using MCFLIRT were used to calculate the frame-wise displacement (FD), i.e. the sum of the absolute derivatives of the 3 translational parameters (x, y and z) and the 3 rotational parameters (yaw α, pitch β and roll γ) converted to distances by computing the arc length displacement on the surface of a sphere with radius 50 mm[2]:

$$FD_{t}=\underset{d\in D}{\sum }\left|d_{t}-d_{t-1}\right|+50\cdot \frac{\pi }{180}\underset{r\in R}{\sum }\left|r_{t}-r_{t-1}\right|$$

where t = {1, …, N}, D = {x, y, z} and R = {α, β, γ}.

The mean relative displacement provided by MCFLIRT[32] was compared between the two groups using a two-sample t-test.

### De-noising approaches

Single-echo data were then further analyzed using six different cleaning approaches:

1. Single-echo Uncleaned (SE-Uncleaned from here): No additional cleaning;
2. Motion, WM and Cerebrospinal fluid (MWC from here) regression[5, 34]: WM and the CSF mean signals were first extracted from each participant’s pre-processed dataset using standard WM and CSF masks co-registered to each individual’s space[35, 36] and eroded in order to minimize the contribution of gray matter partial volume effects. We then regressed out the average WM and CSF signals and the 24 motion parameters, i.e. the six rigid-body parameter time-series, their backward-looking temporal derivatives and the squares of the twelve resulting regressors;
3. FIXsoft [18, 19]: Single-participant spatial ICA with automatic dimensionality estimation was performed in MELODIC [37] followed by ICA-based automatic de-noising using FIX. With the soft option, a linear regression is performed on the full mixing matrix estimated by ICA and containing both good and bad ICs. This method allows to specifically remove the variance assigned to the identified artefactual components [11]. The full variance of the 24 motion parameters is also regressed out;
4. FIX aggressive (FIXagg)[18, 19]: Single-participant spatial ICA with automatic dimensionality estimation was performed in MELODIC [37] followed by ICA-based automatic de-noising using FIX. With the aggressive option, the bad ICs are fully regressed out of the data, which means that all variance associated with these artefactual components is removed, including the shared variance with good ICs [11]. The full variance of the 24 motion parameters is also regressed out.
  Of note, the FIX training dataset used to discriminate good and bad components for both FIXagg and FIXsoft was acquired from an independent age and sex-matched group of healthy controls (N = 42; 35.7 ± 22.3 years; M/F: 19/23), using an identical ME-EPI sequence. A classification threshold of 5 was chosen to balance between noise removal and signal loss [38–40], obtaining a sensitivity of 96.6% and a specificity of 56.1% on leave-one-out testing.
  We visually confirmed that FIX also successfully identified artifactual components in the ADHD patient data.
5. ICA-AROMA soft (ICA-AROMAsoft)[11]: Single-participant spatial ICA with automatic dimensionality estimation was performed in MELODIC [37] followed by ICA-based de-noising using ICA-AROMA. With the soft option, also called ‘non-aggressive’ by its developers, a linear regression is performed on the full mixing matrix estimated by ICA and containing both good and bad ICs. This method allows to specifically remove the variance assigned to the identified artefactual components [11].
6. ICA-AROMA aggressive (ICA-AROMAagg)[11]: Single-participant spatial ICA with automatic dimensionality estimation was performed in MELODIC [37] followed by ICA-based de-noising using ICA-AROMA. With the aggressive option, the bad ICs are fully regressed out of the data, which means that all variance associated with these artefactual components is removed, including the shared variance with good ICs [11].
  After all these de-noising approaches, data were high-pass temporal filtered with a cut-off frequency of 0.01 Hz, as suggested by their developers.
  The multi-echo data were further analyzed using three different cleaning approaches:
7. ME-Uncleaned: The OC dataset pre-processed with AFNI was high-pass temporal filtered with FSL to allow comparison of SE-Uncleaned and ME-Uncleaned images;
8. ME-ICA: the OC data were cleaned with the AFNI tool *meica.py*[20, 27]. Multi-echo principal components analysis was first applied to the OC dataset to reduce the data dimensionality. Spatial ICA was then applied and the independent component time-series were fit to the pre-processed time-series from each of the three echoes to generate ICA weights for each echo. These weights were then fit to the linear TE-dependence and TE-independence models to generate F-statistics and component-level κ and ρ values, which respectively indicate BOLD and non-BOLD weightings. The κ and ρ metrics were then used to identify non-BOLD-like components to be regressed out of the OC dataset as noise regressors. Further technical details on ME-ICA can be found in[41].
9. ME-AROMAagg: we also used ICA-AROMAagg to clean-up the OC dataset in order to compare one of the single-echo de-noising methods to ME-ICA and see whether similar results were obtained using the multi-echo sequence, regardless of the ICA-based algorithm used. We chose ICA-AROMA as it is the most similar to ME-ICA (both ICA-AROMA and ME-ICA are ICA-based and able to work autonomously, without training a classifier). Single-participant spatial ICA with automatic dimensionality estimation was performed in FEAT followed by ICA-based de-noising using ICA-AROMA (full component regression).
  Of note, all the ICA-based methods included a first standard step of dimensionality reduction performed by PCA [37].

A study-specific template representing the average T1-weighted anatomical image across ADHD and control groups was built using the Advanced Normalization Tools (ANTs)[42] toolbox. Each participant’s cleaned dataset was co-registered to its corresponding structural scan, then normalized to the study-specific template before warping to standard MNI152 space, with 2×2×2mm^3^ resampling.

### Data quality estimation

The effects of each of the cleaning procedures on data quality for both participant groups were evaluated using a comprehensive range of measures, including: DVARS[2, 24], temporal SNR[19, 20, 22, 25], power spectral density[19] and loss in temporal degrees of freedom associated with each cleaning procedure[7].

Head movement was quantified using DVARS, i.e. the frame-to-frame root mean square change in BOLD signal. DVARS measures the amount of intensity change between consecutive time-points and is estimated by differentiating the volumetric time-series, then calculating the RMS signal change over the whole brain[2, 24]. For t = {1, …, N}, where N is the number of time points, DVARS was calculated as follows:

$$DVARS_{t}=\sqrt{\frac{1}{V}\sum_{I\in V}\langle {\left(I_{t}-I_{t-1}\right)}^{2}\rangle }$$

Where V is the total number of non-null voxels.

DVARS was assessed for each participant for each cleaning method before co-registration to the standard space. DVARS standard deviation across time-points was estimated at the individual participant level. Of note, this measure was computed after grand mean scaling, to compensate for potential scaling differences between SE and ME data.

Temporal SNR was estimated by dividing the mean image intensity across time by its standard deviation over time[19]. The median temporal SNR value across the subject-specific grey matter volume was calculated for each subject.

As the brain stem is a particularly relevant area known to be highly sensitive to noise [43], we also specifically studied the temporal SNR within this area by extracting for each subject and method the mean temporal SNR within a mask of the brain stem co-registered to native space and comparing the methods with a paired t-test.

These two metrics (DVARS and temporal SNR) are expected to show some degree of correlation, as they both measure the temporal variability of the data. Nevertheless, we used both as DVARS tends to be more sensitive to the presence of sudden peaks between contiguous volumes.

We additionally extracted subject-specific RSNs for each cleaned dataset using template-based dual regression[44] using ten RSNs[45] as common spatial regressors. We then used the subject-specific RSN time-series to estimate the mean power spectrum of each cleaned dataset. For each group, the power spectrum was obtained by scaling the cleaned time-series of each RSN by the standard deviation of the corresponding SE-Uncleaned time-series, averaging the spectra across subjects and calculating the median across RSNs[19].

We further evaluated the loss in temporal degrees of freedom associated with each of the different cleaning approaches to index their potential impact on statistical power. The subject-specific number of lost temporal degrees of freedom was estimated considering all nuisance regressors and/or independent components regressed out of the data. We also accounted for the high-pass temporal filtering for the single-echo cleaning approaches. The total number of available temporal degrees of freedom was expressed as the total number of volumes within the rfMRI time-series[7].

Data from DVARS standard deviation, the temporal SNR and the loss in temporal degrees of freedom were tested for normal distribution with the Kolmogorov-Smirnov test. Wilcoxon signed-rank tests were used to compare the non-normally distributed data between the cleaning methods separately for the two groups. In order to perform a fair comparison between single-echo and multi-echo methods, each data quality metric was also tested considering both the filtered and the unfiltered single-echo data.

### Ability to uncouple BOLD signal and head motion

For each data cleaning approach, rfMRI time-series were extracted from 160 regions of interest (ROIs)[46], using a 10-mm sphere centered on the seed coordinates. Functional connectivity among these regions was then estimated and expressed as Z-score. The correlation between ROI-to-ROI FC values and mean relative displacements was estimated across subjects and transformed to a Z-score[7]. Results were finally tested for normal distribution with the Kolmogorov-Smirnov test and compared between data cleaning procedures separately for the two groups.

In order to qualitatively evaluate the amount of FC removed from the pre-processed, uncleaned images because incorrectly identified as spurious by each de-noising method, we also estimated the ‘spurious ROI-to-ROI FC matrices’ by subtracting the de-noised matrices from the SE-Uncleaned ones for each subject and method. We also calculated the correlation of these matrices with motion.

Specifically for the ICA-AROMAsoft and ICA-AROMAagg de-noising methods, in order to evaluate the effect of slice timing correction on the uncoupling between FC and motion we also estimated the ROI-to-ROI FC maps for the slice-timing corrected datasets and their correlation with the mean relative displacement.

We also tested whether Euclidean distance between nodes biases FC scores, resulting in an increased FC between short-range connections and a decreased FC between long-range connections. FC distribution between nodes was expressed in relation to the Euclidean distance between their centers. The group-level ΔR[2], i.e. the difference in FC between de-noised data and SE-Uncleaned data, was estimated for each method. For each de-noising method, the group-level ΔR plot was obtained by computing ΔR for all the pair-wise correlations between the 160 ROIs at a single-subject level, averaging the ΔR vectors across all subjects and plotting ΔR as a function of the Euclidean distance between the centers of these regions.

### Ability to preserve the BOLD signal

The posterior cingulate cortex (PCC), a cortical component of the DMN[47–50], was chosen as the region of interest (ROI). In accordance with previous studies[22, 51, 52], we created a 6-mm radius sphere centered on the MNI coordinates x = 0, y = -54, z = 26, and extracted the corresponding mean time-series from each participant’s dataset. Seed-based voxel-wise FC maps were then obtained by computing the linear correlation between the PCC time-series and the time-series of voxels across the whole brain using REST[53]. Correlation maps were then converted to Z-maps using the Fisher's transformation and qualitatively compared across the different cleaning procedures.

To quantitatively define the differences between the de-noising procedures, we also extracted the z-FC values in the peaks of interest, i.e. the medial prefrontal cortex (mPFC), the left and the right inferior parietal lobules (IPL) and the left and the right hippocampi, for each subject and method. The peaks were identified by using the mean z-FC map across subjects and methods.

These FC values were then tested for normal distribution with the Kolmogorov-Smirnov test and compared between data cleaning procedures separately for the two groups.

## Results

### Data quality

The ADHD group showed significantly greater head movement (mean relative displacement) compared to the HC group (mean_ADHD_ = 0.11 ± 0.1; mean_HC_ = 0.06 ± 0.05; t-value = 2.04; p = 0.004, see also S1 Fig).

**Fig 1:**
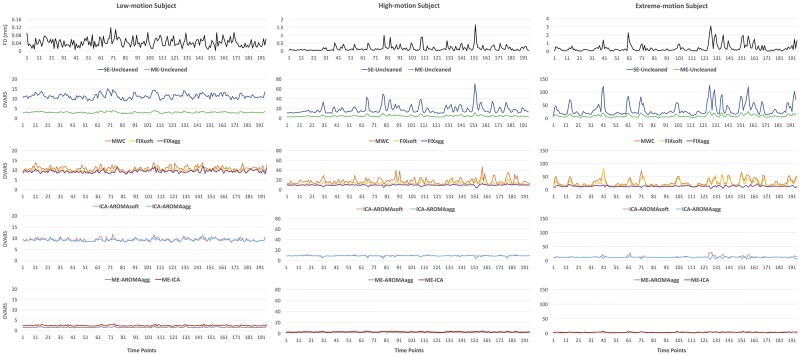
Example of participants with different degrees of head motion: Low motion on the left (mean relative displacement = 0.03 mm), high motion in the central column (mean relative displacement = 0.13 mm) and extreme motion on the right (mean relative displacement = 0.39 mm). From top to bottom the traces of: Frame-wise displacement (FD); comparison of DVARS for data cleaned using SE-Uncleaned (blue line), ME-Uncleaned (green line), MWC (orange line), FIXsoft (yellow line), FIXagg (purple line), ICA-AROMAsoft (pink line), ICA-AROMAagg (light-blue line), ME-AROMAagg (red line) and ME-ICA (grey line). Left, central and right plots are differently scaled in order to have a good view of the low-motion subject’s lines. The DVARS related to each data cleaning procedure qualitatively shows that for the low motion subject (left) all the methods work efficiently, while for the high motion subjects (middle and right) MWC and FIXsoft do not effectively remove most motion-related peaks. FIXagg, ICA-AROMAsoft and ICA-AROMAagg provide better results compared to the other single-echo methods, but the best results were obtained with ME-AROMAagg and ME-ICA. In fact, their DVARS traces are flattened and show a substantial reduction of the motion-related peaks.

FD and cleaning-specific DVARS for three subjects, one exhibiting low head motion (HC, mean relative displacement = 0.03), one exhibiting high head motion (ADHD, mean relative displacement = 0.13) and one exhibiting extreme head motion (ADHD, mean relative displacement = 0.39) are reported in Fig 1 as a qualitative example of the efficiency in removing peaks related to motion. It is important to highlight that the head motion case reported in the figure on the right is quite extreme. However, it is fairly typical for ADHD populations [54, 55]. The figure shows that all the cleaning methods efficiently removed the intensity peaks for the subject with low head motion, while residual peaks, albeit of lower amplitude, are still present after FIXsoft and MWC in the higher motion subjects. FIXagg, AROMAsoft, ICA-AROMAagg, ME-AROMAagg and ME-ICA have a strong impact on signal variation, reducing the peak height to less than 10% of their original value.

**Fig 2:**
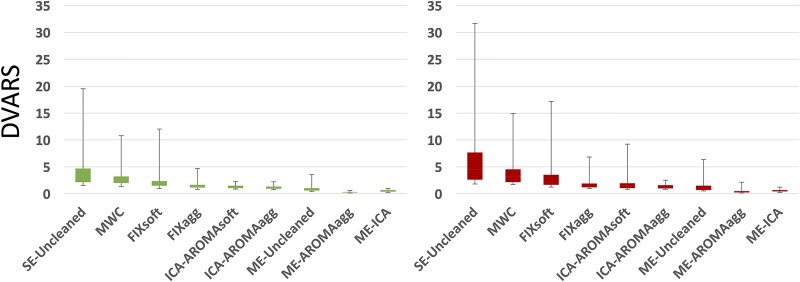
DVARS standard deviation (variability) for the data cleaning methods and for HC (green) and ADHD (red) groups. FIXagg, ICA-AROMAsoft and ICA-AROMAagg provide better results compared to MWC and FIXsoft. However, ME-AROMAagg and ME-ICA results show the greatest reduction of variability for both groups. S1 and S2 Tables respectively present DVARS variability values (mean ± standard deviation) for each method and results obtained from the comparison (Wilcoxon signed-rank test) between the methods.

The DVARS standard deviation at the individual participant level for the two groups with the different cleaning options is shown in Fig 2. Comparison of methods was performed using the Wilcoxon signed-rank test, as the data were non-normally distributed. All the comparisons highlighted significant differences between the data cleaning methods (|z| = 1.985÷4.782, p<0.05) except for the pairs FIXagg-ICA-AROMAsoft (z_ADHD_ = 0.53, p = n.s), ICA-AROMAagg-ME-Uncleaned (z_ADHD_ = 0.792, p = n.s) ME-AROMAagg-ME-ICA (z_ADHD_ = 1.882, p = n.s.). Considering both single-echo and multi-echo cleaning methods, FIXagg, ICA-AROMAsoft, ICA-AROMAagg, ME-Uncleaned and ME-ICA provided the lowest mean values, indicating a more efficient removal of sudden intensity changes related to head movement, while ME-AROMAagg minimized the standard deviation of DVARS, indicating the strongest impact on signal variability. DVARS variability values (mean ± standard deviation) and results obtained from the statistical comparison between the methods are respectively reported in S1 and S2 Tables. The results of this comparison for unfiltered single-echo data were very similar to those obtained using filtered data in terms of statistical significance, except for the pairs AROMAagg-ME-ICA (HC group, non-significant p-value) and FIXagg-ME-Uncleaned (z_HC_ = 2.705, p = 0.007).

**Fig 3:**
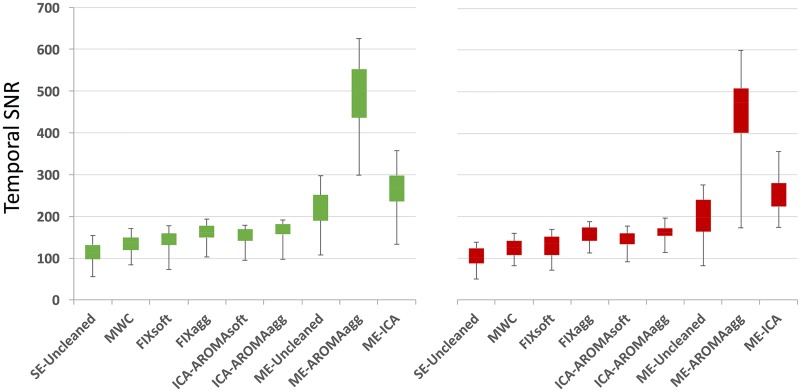
Temporal SNR estimation for every cleaning procedure and for HC (green) and ADHD (red) groups. For each subject and cleaning approach, a temporal SNR image was created by dividing the mean image across time by the standard deviation image over time. The median value across subject-specific gray matter volume was then calculated to represent the temporal SNR value. The boxplots show the temporal SNR value across subjects for various cleaning procedures in the two groups. ME-Uncleaned, ME-AROMAagg and ME-ICA temporal SNRs were scaled dividing by the square root of 3, in order to adjust for the different number of images per time-point. S3 and S4 Tables respectively present the temporal SNR variability values (mean ± standard deviation) for each method and the results obtained from the comparison (Wilcoxon signed-rank test) between the methods.

With respect to temporal SNR, all comparisons between the data cleaning methods highlighted significant differences (|z| = 2.705÷4.782, p≤0.007), except for the pair FIXagg—ICA-AROMAagg in both groups (z_HC_ = 1.018, z_ADHD_ = 1.039, p = n.s.). Of note, ME-Uncleaned, ME-AROMAagg and ME-ICA temporal SNRs were scaled by dividing by the square root of 3 to adjust for the higher number of images per time-point. ME-ICA and ME-AROMAagg temporal SNRs were significantly higher than all the single-echo methods and ME-Uncleaned (mean and standard deviation of each method are reported in the S3 Table). ME-AROMAagg provided the highest temporal SNR values, despite the penalizing rescaling. These data are summarized in Fig 3; results obtained from the comparison between the methods are reported in the S4 Table. Again, the results obtained with unfiltered single-echo data were similar in terms of statistical significance, except for the pair FIXsoft-ICA-AROMAsoft (ADHD group, non-significant p-value). The analysis of tSNR within the brain stem showed similar results to those found considering the whole grey matter (S2 Fig). Results obtained from the comparison between the methods are reported in the S5 Table.

**Fig 4:**
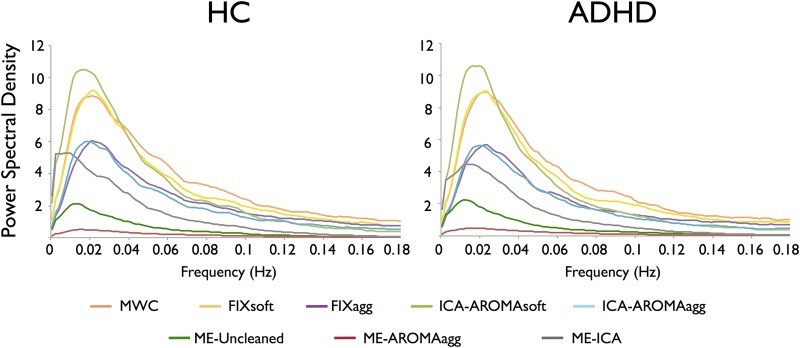
Power spectral density plotted for every data cleaning strategy, per dataset. ICA-AROMAsoft, MWC and FIXsoft showed the highest power spectral density amplitudes. MWC and FIXsoft also showed residual power in the non-BOLD frequencies. FIXagg and ICA-AROMAagg exhibited a power spectral density reduction at high frequencies and a drop of the very low frequencies. ME-ICA cleanup efficiently reduced the power spectral density variability, suppressed the high frequencies and preserved the ultra-slow frequencies. ME-Uncleaned and ME-AROMAagg power spectral densities were very flattened at low and high frequencies.

Regarding the power spectral densities (Fig 4), MWC and FIXsoft showed very high power spectral density amplitudes and residual power in the high frequency ranges (typically non-BOLD frequencies). FIXagg and ICA-AROMAagg showed a power spectral density reduction at high frequencies and a drop of very low frequencies related to the high-pass filter cut-off at 0.01 Hz. Of note, ICA-AROMAsoft provided the highest power spectral density amplitudes and a power spectral density reduction at high frequencies similar to FIXagg and ICA-AROMAagg. Regarding the multi-echo methods, ME-ICA cleanup efficiently reduced the power spectral density amplitude, suppressed high frequencies while simultaneously preserving very low frequencies. Conversely, ME-AROMAagg drastically reduced not only the high frequencies, but also those in the BOLD range.

**Fig 5:**
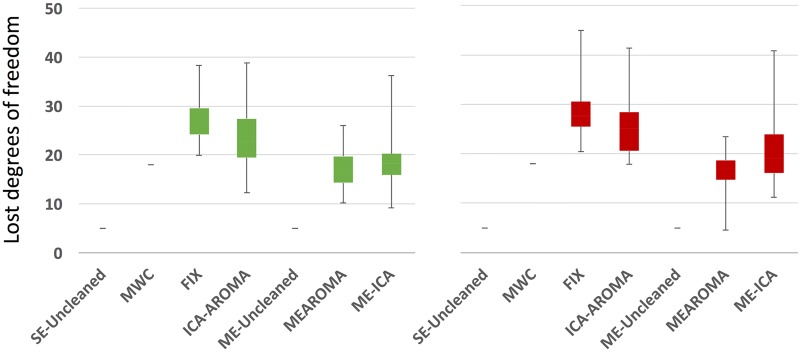
Percentage of the lost temporal degrees of freedom for every cleanup strategy, per dataset (HC in green, ADHD in red). Total available temporal degrees of freedom were expressed as the available number of time-points.

The percentage loss in temporal degrees of freedom is represented in Fig 5, while the number of estimated and removed components for the ICA-based methods is reported in S3 Fig. SE-Uncleaned and ME-Uncleaned methods were affected by high-pass filtering only. Comparing the ICA-based methods using Wilcoxon signed-rank test, it emerged that the loss in temporal degrees of freedom associated with the multi-echo methods was significantly lower compared to the single-echo ones (p<0.05). Of note, ICA-AROMAagg and ME-AROMAagg provided a different number of components too. In S4 Fig we reported an example of good components classified by the two approaches for the high-motion subject whose FD and DVARS parameters have been reported in Fig 1.

As regards the multi-echo methods, the ME-AROMAagg loss in temporal degrees of freedom was significantly lower compared to ME-ICA (Z_HC_ = 2.236, p_HC_ = 0.025; Z_ADHD_ = 2.358, p_ADHD_ = 0.018). Considering the unfiltered single-echo data, results showed significant differences for FIX>ICA-AROMA (z_HC_ = 2.87, p = 0.004; z_ADHD_ = 3.182, p = 0.001), FIX>ME-AROMA (z_HC_ = 4.155, p = 0.001; z_ADHD_ = 4.66, p<0.001), FIX>ME-ICA (z_HC_ = 2.79, p = 0.005), ICA-AROMA>ME-AROMA (z_ADHD_ = 3.144, p = 0.002) and ME-ICA>ME-AROMA (z_HC_ = 2.236, p = 0.025; z_ADHD_ = 2.358, p = 0.018).

### Ability to uncouple BOLD signal and head motion

**Fig 6:**
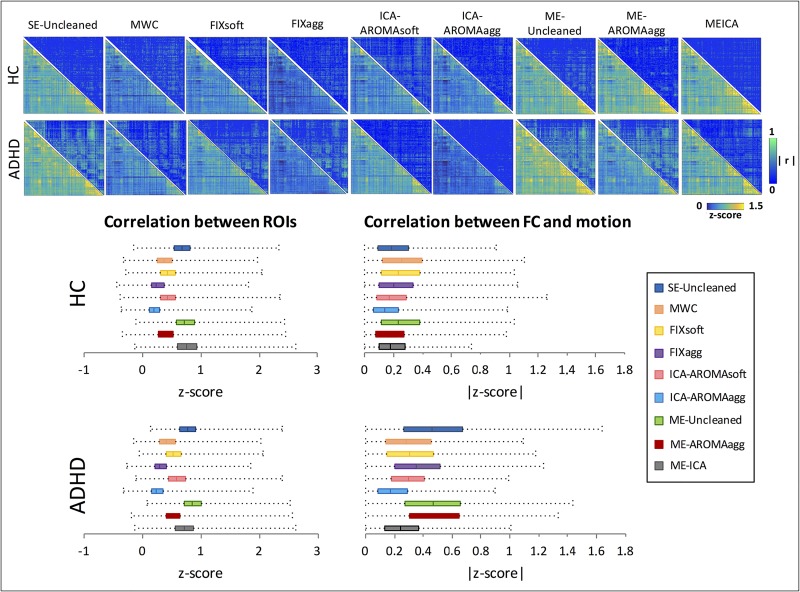
Motion artifact removal in ROI-based functional connectivity analysis (160 ROIs). On the top, the lower triangular matrices show the group-averaged FC matrices (Fisher R-to-Z transformed) for each cleaning procedure (HC subjects in the first row, ADHD patients in the second one). The upper triangular matrices show the correlation across subjects between the ROI-to-ROI FC and the mean relative displacement. On the bottom, the distributions of the FC scores (left) and of the correlation between FC and motion expressed in Z-score (right) are displayed. The SE-Uncleaned data show a low degree of correlation between FC and motion for the HC group, and a high degree of correlation for the ADHD group. Taking those results as a basis for comparison, it can be observed that, in the HC group, ME-AROMAagg and ME-ICA preserve the FC structure (lower triangles) without increasing the correlation with motion (upper triangles). In the ADHD group, ME-ICA and ICA-AROMAsoft minimize the correlation between FC and motion, while preserving the FC score range, while ME-AROMAagg is inefficient in removing the FC correlation with motion. MWC, FIXsoft and FIXagg do not show effective motion artifact removal. ICA-AROMAagg seems to impact on the correlation between FC and motion in the ADHD subjects, but is also shows a substantial reduction of FC values. Results obtained from the comparison of FC values and from correlations between FC and relative motion displacement between methods are reported in Tables 1 and 2 respectively (Wilcoxon signed-rank tests).

The ability of each method to uncouple BOLD signal and head motion and concurrently preserve the signal of interest was assessed by evaluating FC structure and correlating the ROI-to-ROI FC values with the mean relative displacement across participants. Fig 6 shows the mean FC matrix for each group and cleaning procedure (lower triangles) and their respective motion-dependent correlation matrices (upper triangles). The mean FC values and correlations between FC and motion were arranged in vectors and compared using Wilcoxon signed-rank test (Tables 1 and 2). We expect an efficient cleaning method to minimize the correlation value in the upper triangles and to preserve the correlation structure in the lower triangle, as explained in[7]. In the HC group, SE-Uncleaned data were adequately uncoupled with motion. Compared to the SE-Uncleaned data, all the single-echo methods showed a significant decrease of FC between the seed-ROI pairs. MWC, FIXsoft and FIXagg also introduced a certain amount of coupling with motion, while ME-ICA and ME-AROMAagg preserved the uncoupling. By comparison, in the ADHD group, SE-Uncleaned data showed a greater correlation between motion and seed-pair correlation scores, due to the more extensive degree of head movement. Consistently, higher residual correlations between FC and motion can be observed for all the de-noising methods compared to the HC group. Nevertheless, the statistical results indicated that all the single and multi-echo methods, except for ME-AROMAagg, significantly reduced the coupling between FC and motion compared to SE-Uncleaned data (Table 2). However, as can be seen from Fig 6, almost all the single-echo methods exhibited either insufficient cleanup expressed by a high residual coupling of FC with motion in the upper triangular matrices of MWC, FIXsoft, FIXagg, and/or a drastic impact on FC correlation structure, showed by the strong reduction of FC in the lower triangular matrices of MWC, FIXagg, ICA-AROMAagg. By contrast, ME-ICA and ICA-AROMAsoft are the only methods that returned an almost complete uncoupling between FC and mean relative displacement and a concurrent preservation of the correlations between pair-wise regional time series.

**Table 1:** Comparison of FC values among different cleaning approaches (Wilcoxon signed-rank test).

	HC		ADHD	
	Z	p-value	Z	p-value
SE-Uncleaned < MWC	97.675	<0.001	97.675	<0.001
SE-Uncleaned < FIXsoft	97.674	<0.001	97.669	<0.001
SE-Uncleaned < FIXagg	97.675	<0.001	97.675	<0.001
SE-Uncleaned < ICA-AROMAsoft	97.600	<0.001	96.897	<0.001
SE-Uncleaned < ICA-AROMAagg	97.675	<0.001	97.675	<0.001
SE-Uncleaned < ME-Uncleaned	-63.395	<0.001	-83.176	<0.001
SE-Uncleaned < ME-ICA-AROMAagg	97.612	<0.001	97.471	<0.001
SE-Uncleaned < ME-ICA	-73.978	<0.001	53.224	<0.001
MWC < FIXsoft	-75.133	<0.001	-95.289	<0.001
MWC < FIXagg	94.355	<0.001	91.026	<0.001
MWC < ICA-AROMAsoft	-60.676	<0.001	-96.936	<0.001
MWC < ICA-AROMAagg	96.631	<0.001	95.700	<0.001
MWC < ME-Uncleaned	-97.675	<0.001	-97.675	<0.001
MWC < ME-ICA-AROMAagg	-23.176	<0.001	-77.959	<0.001
MWC < ME-ICA	-97.674	<0.001	-97.664	<0.001
FIXsoft < FIXagg	97.675	<0.001	97.675	<0.001
FIXsoft < ICA-AROMAsoft	0.764	0.445	-60.818	<0.001
FIXsoft < ICA-AROMAagg	97.672	<0.001	97.674	<0.001
FIXsoft < ME-Uncleaned	-97.635	<0.001	-97.622	<0.001
FIXsoft < ME-ICA-AROMAagg	35.286	<0.001	6.148	<0.001
FIXsoft < ME-ICA	-97.675	<0.001	-96.187	<0.001
FIXagg < ICA-AROMAsoft	-97.514	<0.001	-97.675	<0.001
FIXagg < ICA-AROMAagg	81.662	<0.001	84.439	<0.001
FIXagg < ME-Uncleaned	-97.675	<0.001	-97.675	<0.001
FIXagg < ME-ICA-AROMAagg	-96.691	<0.001	-97.560	<0.001
FIXagg < ME-ICA	-97.675	<0.001	-97.675	<0.001
ICA-AROMAsoft < ICA-AROMAagg	97.675	<0.001	97.675	<0.001
ICA-AROMAsoft < ME-Uncleaned	-97.334	<0.001	-96.965	<0.001
ICA-AROMAsoft < ME-ICA-AROMAagg	33.486	<0.001	51.246	<0.001
ICA-AROMAsoft < ME-ICA	-97.669	0.417	-92.736	<0.001
ICA-AROMAagg < ME-Uncleaned	-97.675	<0.001	-97.675	<0.001
ICA-AROMAagg < ME-ICA-AROMAagg	-97.643	<0.001	-97.674	<0.001
ICA-AROMAagg < ME-ICA	-97.675	<0.001	-97.675	<0.001
ME-Uncleaned < ME-ICA-AROMAagg	97.662	<0.001	97.660	<0.001
ME-Uncleaned < ME-ICA	-35.083	<0.001	85.652	<0.001
ME-ICA-AROMAagg < ME-ICA	-97.675	<0.001	-92.770	<0.001

**Table 2:** Comparison of FC-motion uncoupling among different cleaning approaches (Wilcoxon signed-rank test).

	HC		ADHD	
	Z	p-value	Z	p-value
SE-Uncleaned < MWC	38.562	<0.001	-66.315	<0.001
SE-Uncleaned < FIXsoft	25.211	<0.001	-60.117	<0.001
SE-Uncleaned < FIXagg	10.437	<0.001	-44.771	<0.001
SE-Uncleaned < ICA-AROMAsoft	-8.251	<0.001	-70.443	<0.001
SE-Uncleaned < ICA-AROMAagg	-28.505	<0.001	-84.502	<0.001
SE-Uncleaned < ME-Uncleaned	31.655	<0.001	-1.995	0.046
SE-Uncleaned < ME-ICA-AROMAagg	-13.970	<0.001	-.503	0.615
SE-Uncleaned < ME-ICA	-7.843	<0.001	-73.918	<0.001
MWC < FIXsoft	-9.263	<0.001	-5.407	<0.001
MWC < FIXagg	-30.529	<0.001	-27.970	<0.001
MWC < ICA-AROMAsoft	-39.629	<0.001	-7.072	<0.001
MWC < ICA-AROMAagg	-54.256	<0.001	-53.733	<0.001
MWC < ME-Uncleaned	-9.187	<0.001	-60.409	<0.001
MWC < ME-ICA-AROMAagg	-43.569	<0.001	-69.497	<0.001
MWC < ME-ICA	-45.559	<0.001	-22.203	<0.001
FIXsoft < FIXagg	-20.383	<0.001	-29.287	<0.001
FIXsoft < ICA-AROMAsoft	-31.224	<0.001	-12.961	<0.001
FIXsoft < ICA-AROMAagg	-48.122	<0.001	-58.111	<0.001
FIXsoft < ME-Uncleaned	-0.199	0.842	-58.465	<0.001
FIXsoft < ME-ICA-AROMAagg	-37.089	<0.001	-66.286	<0.001
FIXsoft < ME-ICA	-34.022	<0.001	-25.860	<0.001
FIXagg < ICA-AROMAsoft	-18.318	<0.001	-36.301	<0.001
FIXagg < ICA-AROMAagg	-39.424	<0.001	-76.544	<0.001
FIXagg < ME-Uncleaned	15.238	<0.001	-40.638	<0.001
FIXagg < ME-ICA-AROMAagg	-24.979	<0.001	-51.046	<0.001
FIXagg < ME-ICA	-21.307	<0.001	-43.976	<0.001
ICA-AROMAsoft < ICA-AROMAagg	-31.070	<0.001	-72.434	<0.001
ICA-AROMAsoft < ME-Uncleaned	32.260	<0.001	-68.835	<0.001
ICA-AROMAsoft < ME-ICA-AROMAagg	-5.520	<0.001	-75.815	<0.001
ICA-AROMAsoft < ME-ICA	0.811	0.417	-19.613	<0.001
ICA-AROMAagg < ME-Uncleaned	47.253	<0.001	-84.161	<0.001
ICA-AROMAagg < ME-ICA-AROMAagg	16.685	<0.001	-89.198	<0.001
ICA-AROMAagg < ME-ICA	25.684	<0.001	-34.503	<0.001
ME-Uncleaned < ME-ICA-AROMAagg	-36.968	<0.001	-1.809	0.07
ME-Uncleaned < ME-ICA	-33.364	<0.001	-74.492	<0.001
ME-ICA-AROMAagg < ME-ICA	7.801	<0.001	-77.544	<0.001

We also reported the spurious ROI-to-ROI FC matrices and their correlation with motion in S5 Fig. These FC matrices complementarily show that the amount of FC classified as spurious (i.e., led by artefactual sources instead of a true functional relationship between the ROIs) by FIXsoft and ICA-AROMAagg is higher compared to all the other methods. Moreover, this incorrect classification is evident both in the high-motion population and in the low-motion one, but the removal of such great amount of spurious FC in HC is not reflected by a concurrent great reduction of correlation with motion.

The comparison between ICA-AROMAsoft and ICA-AROMAagg matrices obtained from data pre-processed with and without the slice timing correction is reported in S6 Fig. The figure shows small differences between the slice timing corrected and the non-corrected datasets in terms of FC. However, the corrected datasets also show a higher residual coupling between FC and motion compared to the respective non-corrected datasets.

We also studied the relationship between ROI-to-ROI FC and their Euclidean distance, expecting negative ΔR values for short-range connections and positive ΔR values for long-range ones. This would mean that the de-noising methods decrease the known bias related to inter-nodal distance[2], reducing spurious correlations between close nodes and increasing FC between distant nodes. S7 Fig shows that a dependency exists between ROI-to-ROI FC and distance for the SE-Uncleaned data, especially in the ADHD dataset (red scatter plot). Looking at the ΔR scatter plots of the ADHD group (in yellow), almost all methods reported a positive linear trend. However, most single-echo methods and ME-AROMAagg show negative ΔR values throughout. This indicates that FC reduction involves not only the short-range connections, which are the most affected by this FC decrease, but also the long-range ones. The linear trend showed by ME-ICA shows instead negative ΔR values for the short connections, positive ΔR values for the distant connections and ΔR = 0 for the medium-range ones, i.e. a reduction of the short-range FC values and an increase of the long-range ones.

### Ability to preserve the BOLD signal

**Fig 7:**
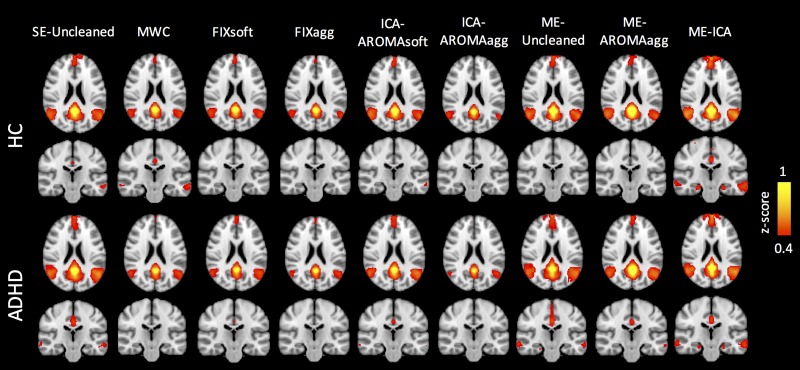
Quality of seed-based Functional Connectivity (FC) in the Default Mode Network (DMN): Comparison of the different de-noising methods. Seed-based FC z-maps, averaged for the subjects belonging to the HC (top) and ADHD (bottom) groups. The seed was placed in the posterior cingulate cortex (PCC, MNI coordinates: 0, -54, 26). Choosing an arbitrary correlation threshold (Z-score) of 0.4, only ME-ICA seems able to efficiently preserve the BOLD signal in both groups and show the typical connectivity between PCC and medial prefrontal cortex, PCC and hippocampi, and PCC and inferior parietal lobules.

Fig 7 shows the thresholded seed-based Z-maps averaged across the HC and ADHD groups for each cleanup method, while the unthresholded maps and the box-plots of the FC values in the DMN peaks are reported in S8 and S9 Figs. As shown in the literature, activity within the mPFC, left and right IPLs and bilateral hippocampi strongly correlated with that from the PCC. The non-parametric paired t-tests highlighted the following results (see also S6 Table):

- most of the single-echo de-noising methods showed a significantly reduced FC compared to SE-Uncleaned method and multi-echo methods, except for ICA-AROMAsoft;
- the aggressive option of both FIX and ICA-AROMA provided lower values of FC compared to their respective soft versions;
- FIXsoft showed a significantly lower FC than ICA-AROMAsoft in the correlations PCC-mPFC (only ADHD, z = 2.602, p = 0.009), PCC-right IPL (z_HC_ = 3.013, p = 0.003; z_ADHD_ = 2.417, p = 0.016) and PCC-right hippocampus (only HC, z = 2.808, p = 0.005);
- ICA-AROMAsoft did not show significant differences with ME-Uncleaned method and ME-AROMAagg;
- ME-ICA showed higher FC values compared to the single echo methods, including ICA-AROMAsoft (z = 2.17÷4.186, p≤0.03, except for the correlation between PCC and mPFC in ADHD and PCC-right IPL in HC, where non-significant differences were found), ME-Uncleaned method (z = 2.314÷4.103, p≤0.021, except for PCC-right IPL in both groups and PCC-left hippocampus in ADHD) and ME-AROMAagg (z = 2.149÷4.268, p≤0.032, except for PCC-left IPL in ADHD).

Overall, ME-ICA better preserved the high correlations between the PCC and all other DMN areas compared to the other methods. Clean-up performed with the single-echo methods resulted in substantially reduced FC among the DMN areas, except for ICA-AROMAsoft that showed higher FC values.

## Discussion

Here we used data from young healthy subjects, who typically show low head movement, and ADHD patients, whose disorder is intrinsically associated with restlessness. We first aimed to corroborate previous work showing that head motion has a substantial impact on FC analysis, and then to characterize the effects of different cleanup techniques on rfMRI data robustness[3, 5, 7].

We compared methods based on both single- and multi-echo acquisition sequences. More specifically, we examined five single-echo approaches − MWC, based on regression of 24 realignment parameters and mean WM and CSF signals; FIX and ICA-AROMA, either including soft and the aggressive options − and ME-ICA, a novel approach that combines a multi-echo acquisition sequence with ICA. We also used ICA-AROMAagg on the multi-echo data, in order to see whether the optimal combination of three echoes[28] can significantly improve the performance of any ICA-based algorithm and demonstrate that the efficiency of ME-ICA also relies on the ICA analysis of TE dependence[27].

Among the single-echo approaches, FIXagg and ICA-AROMAagg showed the greatest ability to reduce DVARS fluctuations, provided the best temporal SNR, and properly reduced power spectral density at high frequencies. However, ME-AROMAagg and ME-ICA performed significantly better than all the other methods in terms of increased temporal SNR, reduced DVARS and preservation of the temporal degrees of freedom. Regarding the temporal degrees of freedom analysis, it was unsurprising that the ICA-based approaches, namely FIX, ICA-AROMA, ME-AROMAagg and ME-ICA, led to a greater loss in temporal degrees of freedom compared to the other methods. However, it is noteworthy that multi-echo methods lost on average less temporal degrees of freedom than single-echo ICA-based approaches.

Considering the spectral domain, it is well known that spontaneous BOLD oscillations are localized in the lower frequency ranges[56]. We analyzed the RSNs’ spectral power distributions to see which method performed the best reduction of the non-BOLD spectral power, while simultaneously preserving power in the BOLD frequency range. Our findings indicate that ME-ICA reduced spectral energy at high frequencies while preserved the full-frequency spectrum of the BOLD signal (i.e., frequencies below 0.1 Hz) [57, 58].

Although quantitative, the interpretation of these results is not straightforward. In fact, rfMRI data nature does not allow discriminating with absolute certainty the noise-related variability and the BOLD-related one that should be preserved. Nevertheless, as already seen in other studies[19, 20, 25], the measures that we used to evaluate the data quality can be considered useful indicators of how much variance is being removed after the cleaning procedures.

Additionally, we investigated the ability of these methods to minimize the effects of head motion, reduce the FC dependence on the anatomical distance between the brain areas and preserve FC between them. Our results showed a marked ability of ME-ICA to uncouple BOLD signal and head motion even in the ADHD population, while still preserving the FC structure. In contrast, MWC and FIX introduced some spurious correlations between FC and motion in the HC group. They also showed decreased levels of FC among the ROIs and resulted in suboptimal removal of the effects of head motion in the ADHD dataset. Regarding the ICA-AROMA approaches, ICA-AROMAagg showed a noticeable reduction of coupling between FC and motion, which is hypothesized to be mainly related to a substantial drop in the FC values instead of an efficient cleanup, while ICA-AROMAsoft showed good performances in obtaining an almost complete uncoupling between FC and mean relative displacement.

As regards ICA-AROMA applied to the OC multi-echo dataset, our results showed that the combination of the multi-echo rfMRI acquisition and ICA-AROMAagg improved the quality of the BOLD components extracted and better preserved the ROI-to-ROI FC. However, it was ineffective in reducing the high degree of uncoupling between FC and motion in the ADHD group, suggesting that the optimal combination of the three echoes is not enough to improve the ICA-based cleanup.

It is also worth noting that by looking at the results of the multi-echo techniques, the advantages of using a multi-echo acquisition instead of the standard single-echo one are clearly detectable, e.g. the higher temporal SNR, the lower DVARS and the increased contrast in some areas that are typically affected by macro-inhomogeneities, which is obtained by using the optimal combination of the three echoes [59]. However, the comparison between ME-AROMAagg and ME-ICA highlighted the added value of using the TE-dependence and TE-independence models to classify the components instead of using specific features extracted from data.

Our findings showed that ME-ICA performed the best motion reduction on the two populations’ datasets, as it did not highly bias the HC correlations between the nodes and efficiently reduced the spurious correlations between FC and the high degree of motion in the ADHD dataset. The complimentary analysis of the ΔR plots corroborates these results, as it shows that ME-ICA is the only method that did not increase the distance-dependent connectivity biases and properly reduced the FC dependence on the Euclidean distance between the nodes without affecting FC values.

Seed-based analysis highlighted the efficiency of ME-ICA in preserving the signal of interest. In fact, DMN areas[47, 48, 50, 60] preserved their functional connections after ME-ICA cleanup. Conversely, the other methods affected the signal of interest in both groups, notably reducing FC between the pairs PCC-hippocampi, PCC-medial prefrontal cortex and PCC-inferior parietal lobules. This might have a negative impact on a clinical study, namely addressing the FC impairment between DMN nodes as candidate biomarkers of neurological disease.

Of note, the de-noising performance of ME-ICA was not influenced by specifically selecting parameters that would be optimal for ME-ICA versus other de-noising techniques. An analytical model to predict optimal multi-echo parameters a priori also could not be established in this study given its limitation of being conducted at 1.5T. Future study of a general relationship between acquisition parameters and/or raw data attributes (i.e. tSNR) and ME-ICA or other de-noising performance would be beneficial. We note however that multi-echo fMRI parameter selection in this study used the same guidelines as for prior 3.0T studies involving ME-ICA (see S1 Appendix for further information).

Overall, ME-ICA was robust in signal de-noising, outperformed the single-echo methods as to data quality after cleaning (higher temporal SNR, lower DVARS, less temporal degrees of freedom lost) and showed a better ability to preserve the BOLD signal in its full-frequency spectrum. Moreover, the shown reduction in coupling between FC and motion in a population with a hyperactivity disorder is particularly promising, as it might suggest that using ME-ICA the chance of finding spurious between-group differences biased by motion is likely to be strongly reduced. We did not perform such a comparison here, because the interpretation of the results in the context of evaluating the performance of cleaning methods would be extremely difficult. The potential bias introduced by motion in group comparisons involving clinical populations characterized by restlessness has been shown before[61]. However, in the absence of an absolute gold-standard, it is impossible to state whether any difference (or indeed lack of difference) is linked to the diagnostic status of this sample, or to motion-related artefacts.

As a general limitation of this work, and of all studies attempting to compare methods of artifact-removal for rfMRI, we would like to reiterate that such comparisons cannot be tested against the ground truth, and therefore any conclusion should be interpreted with caution. As a consequence, we can only conclude that, with respect to the specific metrics used here, which assess some of the potential effects of motion of rfMRI signal, ME-ICA provided, overall, the best performance. We would also like to reiterate that for each method we chose to follow the pre-processing pipeline suggested by their developers. This may have resulted in small differences in the pre-processing, which might have partially biased the comparison. These effects are, however, likely to be very small. Altering the recommended pipeline might have introduced a different form of bias, as we would have had to follow a sub-optimal approach for some of the methods. This was confirmed by comparing the results obtained when including slice-timing correction in the pre-processing of single-echo data before ICA-AROMA. If anything, our data (S5 Fig) suggest that a slightly worse decoupling between FC and motion is achieved when performing slice-timing correction. By using slice-timing correction for both single- and multi-echo datasets, the comparison outcomes would not have changed, but the performances of the single-echo methods would have been penalized.

It is important to emphasize that the quality results shown here were obtained from data acquired using a clinical scanner. This suggests that, if multi-echo EPI sequence were made available on clinical systems, reliable rfMRI data could be obtained in the clinical environment. It would be interesting to evaluate, in further studies, how this de-noising method influences the within-site test-retest reliability and the across-site reproducibility consistency of resting state FC results, as performed by different studies to assess the efficiency of different physiological noise correction techniques [62, 63].

## Conclusion

Our findings confirm that motion is a source of substantial error in rfMRI analysis. They suggest that most of the methods considered here introduce some benefit. Single-echo cleaning methods performed well according to some evaluated parameters but less well for others. Their performance was acceptable in improving data quality and reducing effects of motion on FC in HC. However, all of these methods were suboptimal in removing effects of head motion in the ADHD population, except for ICA-AROMAsoft, which showed good performances for both datasets and can be considered an efficient single-echo de-noising method for high motion populations. However, thanks to integration of multi-echo EPI acquisition of the rfMRI data with ICA, thus distinguishing BOLD from non-BOLD signal components based on relaxometry of their respective and differentiable signatures in the decay domain [59], ME-ICA provided better results in terms of data quality and efficiency to uncouple BOLD signal and motion compared not only to the single-echo methods, but also to ICA-AROMAagg applied to multi-echo data (ME-AROMAagg method). The comparison between single-echo and multi-echo methods confirmed some previous studies[20] and provides additional evidence that ME-ICA is a promising cleaning method for rfMRI data. Going beyond the limitations of the single-echo fMRI and taking advantage of ICA, ME-ICA can be considered a robust data cleaning method that works autonomously (without training any classifier) across scanner platforms[27, 41]. According to the results showed in this paper, studies which involve clinical populations that present greater movement might benefit both from the multi-echo acquisition and the application of ME-ICA for data de-noising.

## Supporting information

S1 Appendix Supplementary methods. (PDF)

S1 File Functional connectivity matrices of the two groups for every de-noising method and mean relative displacement values for all the subjects. (GZ)

S2 File Excel file containing the subject-specific temporal SNR, temporal SNR within the brain stem, DVARS, number of independent components extracted and removed, and the median power spectral density for each method. (GZ)

S3 File Seed-based functional connectivity maps for each HC and ADHD patient for SE-Uncleaned method. (GZ)

S4 File Seed-based functional connectivity maps for each HC and ADHD patient for MWC. (GZ)

S5 File Seed-based functional connectivity maps for each HC and ADHD patient for FIXsoft. (GZ)

S6 File Seed-based functional connectivity maps for each HC and ADHD patient for FIXagg. (GZ)

S7 File Seed-based functional connectivity maps for each HC and ADHD patient for ICA-AROMAsoft. (GZ)

S8 File Seed-based functional connectivity maps for each HC and ADHD patient for ICA-AROMAagg. (GZ)

S9 File Seed-based functional connectivity maps for each HC and ADHD patient for ME-Uncleaned. (GZ)

S10 File Seed-based functional connectivity maps for each HC and ADHD patient for ME-AROMAagg. (GZ)

S11 File Seed-based functional connectivity maps for each HC and ADHD patient for ME-ICA. (GZ)

S1 Table DVARS values for different cleaning approaches. (DOCX)

S2 Table Comparison of DVARS standard deviation among different cleaning approaches (Wilcoxon signed-rank test). (DOCX)

S3 Table Temporal SNR (tSNR) values for different cleaning approaches. The multi-echo temporal SNRs are scaled by dividing by the square root of 3 to adjust for the higher number of images per time-point.
(DOCX)

S4 Table Comparison of temporal SNR among different cleaning approaches (Wilcoxon signed-rank test). (DOCX)

S5 Table Comparison of temporal SNR within the brain stem among different cleaning approaches (Wilcoxon signed-rank test). (DOCX)

S6 Table Comparison of FC between PCC and the other nodes of the DMN among different cleaning approaches. For each comparison, the first and the second rows respectively report the results of HC and ADHD patients.
(DOCX)

S1 Fig Mean motion displacement of the HC (green) and ADHD (red) groups. (TIFF)

S2 Fig Temporal SNR estimation within the brain stem for every cleaning procedure and for HC (green) and ADHD (red) groups. (TIFF)

S3 Fig Number of estimated and removed components for the ICA-based methods. Numbers within the bars indicate the average number of estimated and removed components for each method and group. The whiskers indicate the standard deviation.
(TIFF)

S4 Fig Example of Independent Components (ICs) relative to a high-motion subject and classified as BOLD signal by ICA-AROMAagg and ME-AROMAagg. For this specific subject, ICA-AROMAagg decomposed the signal into 83 ICs and recognized as good only 12 of them (upper panel). Among the good ICs, stripe artifacts are clearly seen in the first three components (IC28, IC57 and IC58), which can still be recognized as resting state networks as include areas respectively belonging to the visual network, executive network and the cerebellum. ME-AROMAagg recognized 22 good ICs out of 66, which is more than twice the good ICA-AROMAagg ICs. However, even if ME-AROMAagg preserved more variance contribution than ICA-AROMAagg, some components (e.g., IC8, IC19, IC42) still presented residual noise.
(TIFF)

S5 Fig Spurious ROI-to-ROI Functional Connectivity (FC) matrix for each method and group and its correlation with motion. Each lower triangular matrix represents the group-averaged difference between the SE-Uncleaned FC matrix and the de-noised one (HC in the first row, ADHD patients in the second one) for each cleaning procedure. The upper triangular matrices show the correlation across subjects between the spurious FC and the mean relative displacement.
(TIFF)

S6 Fig Comparison between the ICA-AROMAsoft and ICA-AROMAagg matrices of FC (lower triangular matrices) and FC-versus-motion (upper triangular matrices) obtained from data pre-processed with (top) and without (bottom) slice timing correction. (TIFF)

S7 Fig Reduction of distance-related functional connectivity biases. The two scatter plots at the top show the dependency of ROI-to-ROI FC on the Euclidean distance between nodes in the SE-Uncleaned data of both groups (green dots for the HC group; red dots for the ADHD group). The ΔR scatter plots on the bottom (blue dots for the HC group; yellow dots for the ADHD group) represent the distribution of the difference in FC between de-noised data and SE-Uncleaned data as a function of the Euclidean distance between the nodes. Single-echo methods and ME-AROMAagg reduced the FC between all nodes in both groups independent of the anatomical distance between them. ME-ICA reduced the short-range functional connections and increased long-range connections in the ADHD group.
(TIFF)

S8 Fig Unthresholded maps of seed-based functional connectivity in the default mode network. These maps are the same of those reported in Fig 7, with the only difference that an arbitrary threshold of the z-score was not applied here.
(TIFF)

S9 Fig Functional connectivity values within the peaks of the regions belonging to the default mode network and correlated with the posterior cingulate cortex (HC in green, ADHD in red). (TIFF)
