# Supplementary material for: Comparing resting state fMRI de-noising approaches using multi- and single-echo acquisitions
Source: PLoS One. 2017 Mar 21;12(3):e0173289. doi: 10.1371/journal.pone.0173289 (PMC5360253; doi:10.1371/journal.pone.0173289)
Supplement: S1 Appendix — (PDF) [file pone.0173289.s001.pdf]

S1 Appendix. Supplementary methods.

Guidelines for acquiring multi-echo rfMRI.

The guidelines optimized for a multi-echo rfMRI sequence in order of priority are:

1. Acquiring at least 3 TEs as minimum for reliable T2* and dT2* fitting;
2. Acquiring an image at the earliest TE allowed by the scanner thus yielding high signal intensity throughout the brain;
3. Acquiring the latest TE image such that most (~75%) of the brain volume has not fully dephases, i.e. most voxels have signal above the noise floor;
4. Acquiring one TE near the mean brain T2* given field strength for having highest single-TE BOLD sensitivity (~60ms at 1.5T, ~30ms at 3.0T);
5. Selecting the minimum GRAPPA factor that allows satisfying conditions (1-4) thus minimizing in-plane acceleration noise - usually GRAPPA 2 up to 3.0T but using SENSE (GE, Phillips) a partial factor between 1-2 also possible;
6. Selecting the minimum TR satisfying (1-5), prioritizing TR<=2.5s in order to sample most of the spectral power of spontaneous BOLD fluctuations;
7. Minimize acquisition bandwidth to decrease Gaussian noise and increase tSNR;
8. Deprioritize spatial resolution towards increasing voxel-wise tSNR, recognizing that:

- with ME-ICA no FWHM smoothing will be applied so both functional sensitivity and effective spatial resolution will be higher than acquiring at high spatial resolution (dictating lower tSNR) natively followed by large-kernel FWHM smoothing (empirical smoothness estimation requires, however).

- dropout reduction sought through acquiring at high spatial resolution in single-echo approaches is accomplished in the multi-echo approach through echo combination leveraging earliest TE signal.

9. Furthermore it is considered that while the need for in-plane acceleration (e.g. GRAPPA, SENSE) is generally considered deleterious but is required here to satisfy (1) and (3), in fact:

- in-plane acceleration decreases spatial distortion and dropout (which are albeit lower at 1.5T than higher field)

- GRAPPA artifacts captured as ICA components are less likely to be as clearly T2*-related and have more S0 weighting than functional BOLD signals so may be removed by ME-ICA as non-neuronal effects. However further study is required to fully characterize BOLD/non-BOLD categorization of partly phase-associated signal sources such as GRAPPA artifacts and draining veins. While acquiring multiple echoes may impart an up-front cost in spatial resolution in an ME experiment compared to an SE experiment, applying standard FWHM spatial smoothing after SE acquisition makes the relative spatial resolution cost of ME acquisition only nominal considering the deleterious impact of FWHM smoothing on multiple comparisons corrections. Choices in this study reflect a consideration that a slightly lower spatial resolution ME scan without FWHM smoothing versus the contrary could impart to an ME approach a decreased penalty for detecting smaller activation fields such as subcortical nuclei (i.e. hippocampus), however further validation is required. As regarding the number of echoes, prior studies have demonstrated use of 3-5 TEs, with higher TE numbers leading particular better estimates of voxel wise T2* from time series means. However, it is vital to remember that ME-ICA leverages the covariance structure of the data to determine signal sources and then utilizes delta-T2* fitting only to determine BOLD/non-BOLD categories, not to directly estimate BOLD time series, which has been shown previously be highly noise-prone. This approach is satisfied by a 3 echo dataset as we have previously demonstrated. Acquiring more than 3 echoes may be beneficial if above conditions can be met, but we considered that major deviations from the above prioritized strategy in pursuit of more echoes had a high

chance of being counterproductive for example by incurring higher GRAPPA artifact penalty for acquiring echoes with low signal amplitude.
