# Supplementary material for: Comparing resting state fMRI de-noising approaches using multi- and single-echo acquisitions
Source: PLoS One. 2017 Mar 21;12(3):e0173289. doi: 10.1371/journal.pone.0173289 (PMC5360253; doi:10.1371/journal.pone.0173289)
Supplement: S1 Table — (DOCX) [file pone.0173289.s013.docx]

**S1 Table**. **DVARS values for different cleaning approaches.**

|  | **HC** | | **ADHD** | |
| --- | --- | --- | --- | --- |
|  | **mean** | **stdev** | **mean** | **stdev** |
| SE-Uncleaned | 17.0 | 7.2 | 19.1 | 13.3 |
| MWC | 15.8 | 4.4 | 17.1 | 7.0 |
| FIXsoft | 13.0 | 4.1 | 14.1 | 6.6 |
| FIXagg | 11.5 | 2.6 | 11.9 | 3.4 |
| ICA-AROMAsoft | 10.7 | 2.1 | 11.0 | 3.3 |
| ICA-AROMAagg | 10.5 | 2.0 | 10.6 | 2.2 |
| ME-Uncleaned | 4.6 | 1.4 | 5.1 | 2.5 |
| ME-AROMAagg | 1.9 | 0.4 | 2.2 | 1.1 |
| ME-ICA | 3.3 | 0.8 | 3.3 | 0.9 |
|  |  |  |  |  |
