# Supplementary material for: Comparing resting state fMRI de-noising approaches using multi- and single-echo acquisitions
Source: PLoS One. 2017 Mar 21;12(3):e0173289. doi: 10.1371/journal.pone.0173289 (PMC5360253; doi:10.1371/journal.pone.0173289)
Supplement: S2 Table — (DOCX) [file pone.0173289.s014.docx]

**S2 Table**. **Comparison of DVARS standard deviation among different cleaning approaches** **(Wilcoxon signed-rank test).**

|  | **HC** | | **ADHD** | |
| --- | --- | --- | --- | --- |
|  | **Z** | **p-value** | **Z** | **p-value** |
| SE-Uncleaned > MWC | 3.198 | <0.001 | 4.330 | <0.001 |
| SE-Uncleaned > FIXsoft | 4.679 | <0.001 | 4.782 | <0.001 |
| SE-Uncleaned > FIXagg | 4.782 | <0.001 | 4.782 | <0.001 |
| SE-Uncleaned > ICA-AROMAsoft | 4.782 | <0.001 | 4.782 | <0.001 |
| SE-Uncleaned > ICA-AROMAagg | 4.782 | <0.001 | 4.782 | <0.001 |
| SE-Uncleaned > ME-Uncleaned | 4.782 | <0.001 | 4.782 | <0.001 |
| SE-Uncleaned > ME-AROMAagg | 4.782 | <0.001 | 4.782 | <0.001 |
| SE-Uncleaned > ME-ICA | 4.782 | <0.001 | 4.782 | <0.001 |
| MWC > FIXsoft | 4.227 | <0.001 | 3.425 | 0.001 |
| MWC > FIXagg | 4.782 | <0.001 | 4.782 | <0.001 |
| MWC > ICA-AROMAsoft | 4.762 | <0.001 | 4.679 | <0.001 |
| MWC > ICA-AROMAagg | 4.782 | <0.001 | 4.782 | <0.001 |
| MWC > ME-Uncleaned | 4.782 | <0.001 | 4.782 | <0.001 |
| MWC > ME-AROMAagg | 4.782 | <0.001 | 4.782 | <0.001 |
| MWC > ME-ICA | 4.782 | <0.001 | 4.782 | <0.001 |
| FIXsoft > FIXagg | 4.741 | <0.001 | 4.782 | <0.001 |
| FIXsoft > ICA-AROMAsoft | 4.412 | <0.001 | 4.659 | <0.001 |
| FIXsoft > ICA-AROMAagg | 4.576 | <0.001 | 4.782 | <0.001 |
| FIXsoft > ME-Uncleaned | 4.782 | <0.001 | 4.782 | <0.001 |
| FIXsoft > ME-AROMAagg | 4.782 | <0.001 | 4.782 | <0.001 |
| FIXsoft > ME-ICA | 4.782 | <0.001 | 4.782 | <0.001 |
| FIXagg > ICA-AROMAsoft | 1.985 | 0.047 | 0.627 | 0.53 |
| FIXagg > ICA-AROMAagg | 3.630 | <0.001 | 3.857 | <0.001 |
| FIXagg > ME-Uncleaned | 4.679 | <0.001 | 3.054 | <0.001 |
| FIXagg > ME-AROMAagg | 4.782 | <0.001 | 4.782 | <0.001 |
| FIXagg > ME-ICA | 4.782 | <0.001 | 4.782 | <0.001 |
| ICA-AROMAsoft > ICA-AROMAagg | 3.610 | <0.001 | 3.671 | <0.001 |
| ICA-AROMAsoft > ME-Uncleaned | 3.692 | <0.001 | 3.157 | 0.002 |
| ICA-AROMAsoft > ME-AROMAagg | 4.782 | <0.001 | 4.782 | <0.001 |
| ICA-AROMAsoft > ME-ICA | 4.782 | <0.001 | 4.782 | <0.001 |
| ICA-AROMAagg > ME-Uncleaned | 2.993 | 0.003 | .792 | 0.428 |
| ICA-AROMAagg > ME-AROMAagg | 4.782 | <0.001 | 4.782 | <0.001 |
| ICA-AROMAagg > ME-ICA | 4.782 | <0.001 | 4.782 | <0.001 |
| ME-Uncleaned > ME-AROMAagg | 4.782 | <0.001 | 4.782 | <0.001 |
| ME-Uncleaned > ME-ICA | 4.782 | <0.001 | 4.782 | <0.001 |
| ME-AROMAagg > ME-ICA | -4.782 | <0.001 | -1.882 | 0.06 |
