# Supplementary material for: Comparing resting state fMRI de-noising approaches using multi- and single-echo acquisitions
Source: PLoS One. 2017 Mar 21;12(3):e0173289. doi: 10.1371/journal.pone.0173289 (PMC5360253; doi:10.1371/journal.pone.0173289)
Supplement: S3 Table — The multi-echo temporal SNRs are scaled by dividing by the square root of 3 to adjust for the higher number of images per time-point. (DOCX) [file pone.0173289.s015.docx]

**S3 Table. Temporal SNR (tSNR) values for different cleaning approaches.** The multi-echo temporal SNRs are scaled by dividing by the square root of 3 to adjust for the higher number of images per time-point.

|  | **HC** | | **ADHD** | |
| --- | --- | --- | --- | --- |
|  | **mean** | **stdev** | **mean** | **stdev** |
| SE-Uncleaned | 113.5 | 22.1 | 101.6 | 28.6 |
| MWC | 134.4 | 19.8 | 124.8 | 22.1 |
| FIXsoft | 141.1 | 23.0 | 129.5 | 27.2 |
| FIXagg | 161.5 | 21.4 | 156.1 | 22.7 |
| ICA-AROMAsoft | 152.5 | 20.5 | 142.7 | 23.0 |
| ICA-AROMAagg | 166.0 | 21.0 | 160.7 | 17.8 |
| ME-Uncleaned | 215.4 | 46.9 | 190.6 | 57.8 |
| ME-AROMAagg | 493.8 | 74.5 | 440.5 | 116.9 |
| ME-ICA | 265.7 | 48.5 | 263.1 | 45.0 |
|  |  |  |  |  |
