# Supplementary material for: Comparing resting state fMRI de-noising approaches using multi- and single-echo acquisitions
Source: PLoS One. 2017 Mar 21;12(3):e0173289. doi: 10.1371/journal.pone.0173289 (PMC5360253; doi:10.1371/journal.pone.0173289)
Supplement: S4 Table — (DOCX) [file pone.0173289.s016.docx]

**S4 Table. Comparison of temporal SNR among different cleaning approaches (Wilcoxon signed-rank test).**

|  | **HC** | | **ADHD** | |
| --- | --- | --- | --- | --- |
|  | **Z** | **p-value** | **Z** | **p-value** |
| SE-Uncleaned < MWC | 4.782 | <0.0001 | 4.782 | <0.0001 |
| SE-Uncleaned < FIXsoft | 4.782 | <0.0001 | 4.782 | <0.0001 |
| SE-Uncleaned < FIXagg | 4.782 | <0.0001 | 4.782 | <0.0001 |
| SE-Uncleaned < ICA-AROMAsoft | 4.782 | <0.0001 | 4.782 | <0.0001 |
| SE-Uncleaned < ICA-AROMAagg | 4.782 | <0.0001 | 4.782 | <0.0001 |
| SE-Uncleaned < ME-Uncleaned | 4.782 | <0.0001 | 4.782 | <0.0001 |
| SE-Uncleaned < ME-AROMAagg | 4.782 | <0.0001 | 4.782 | <0.0001 |
| SE-Uncleaned < ME-ICA | 4.782 | <0.0001 | 4.782 | <0.0001 |
| MWC < FIXsoft | 4.165 | <0.0001 | 2.931 | 0.003 |
| MWC < FIXagg | 4.782 | <0.0001 | 4.782 | <0.0001 |
| MWC < ICA-AROMAsoft | 4.391 | <0.0001 | 4.432 | <0.0001 |
| MWC < ICA-AROMAagg | 4.762 | <0.0001 | 4.782 | <0.0001 |
| MWC < ME-Uncleaned | 4.782 | <0.0001 | 4.618 | <0.0001 |
| MWC < ME-AROMAagg | 4.782 | <0.0001 | 4.782 | <0.0001 |
| MWC < ME-ICA | 4.782 | <0.0001 | 4.782 | <0.0001 |
| FIXsoft < FIXagg | 4.782 | <0.0001 | 4.782 | <0.0001 |
| FIXsoft < ICA-AROMAsoft | 3.198 | 0.001 | 3.301 | 0.001 |
| FIXsoft < ICA-AROMAagg | 4.494 | <0.0001 | 4.762 | <0.0001 |
| FIXsoft < ME-Uncleaned | 4.782 | 0.002 | 4.782 | <0.0001 |
| FIXsoft < ME-AROMAagg | 4.782 | <0.0001 | 4.782 | <0.0001 |
| FIXsoft < ME-ICA | 4.782 | <0.0001 | 4.782 | <0.0001 |
| FIXagg < ICA-AROMAsoft | -2.972 | 0.003 | -2.972 | 0.003 |
| FIXagg < ICA-AROMAagg | 1.018 | 0.309 | 1.039 | 0.299 |
| FIXagg < ME-Uncleaned | 4.556 | <0.0001 | 3.178 | 0.001 |
| FIXagg < ME-AROMAagg | 4.782 | <0.0001 | 4.782 | <0.0001 |
| FIXagg < ME-ICA | 4.762 | <0.0001 | 4.782 | <0.0001 |
| ICA-AROMAsoft < ICA-AROMAagg | 4.782 | <0.0001 | 4.782 | <0.0001 |
| ICA-AROMAsoft < ME-Uncleaned | 4.679 | <0.0001 | 4.021 | <0.0001 |
| ICA-AROMAsoft < ME-AROMAagg | 4.782 | <0.0001 | 4.782 | <0.0001 |
| ICA-AROMAsoft < ME-ICA | 4.762 | <0.0001 | 4.782 | <0.0001 |
| ICA-AROMAagg < ME-Uncleaned | 4.247 | <0.0001 | 2.705 | 0.007 |
| ICA-AROMAagg < ME-AROMAagg | 4.782 | <0.0001 | 4.782 | <0.0001 |
| ICA-AROMAagg < ME-ICA | 4.762 | <0.0001 | 4.782 | <0.0001 |
| ME-Uncleaned < ME-AROMAagg | 4.782 | <0.0001 | 4.782 | <0.0001 |
| ME-Uncleaned < ME-ICA | 4.535 | <0.0001 | 4.782 | <0.0001 |
| ME-AROMAagg < ME-ICA | -4.782 | <0.0001 | -4.618 | <0.0001 |
