# Supplementary material for: Comparing resting state fMRI de-noising approaches using multi- and single-echo acquisitions
Source: PLoS One. 2017 Mar 21;12(3):e0173289. doi: 10.1371/journal.pone.0173289 (PMC5360253; doi:10.1371/journal.pone.0173289)
Supplement: S5 Table — (DOCX) [file pone.0173289.s017.docx]

**S5 Table.** **Comparison of temporal SNR within the brain stem among different cleaning approaches (Wilcoxon signed-rank test).**

|  | **HC** | | **ADHD** | |
| --- | --- | --- | --- | --- |
|  | **Z** | **p-value** | **Z** | **p-value** |
| SE-Uncleaned < MWC | 4.782 | <0.001 | 4.782 | <0.001 |
| SE-Uncleaned < FIXsoft | 4.741 | <0.001 | 4.720 | <0.001 |
| SE-Uncleaned < FIXagg | 4.782 | <0.001 | 4.782 | <0.001 |
| SE-Uncleaned < ICA-AROMAsoft | 4.782 | <0.001 | 4.782 | <0.001 |
| SE-Uncleaned < ICA-AROMAagg | 4.782 | <0.001 | 4.782 | <0.001 |
| SE-Uncleaned < ME-Uncleaned | 4.782 | <0.001 | 4.782 | <0.001 |
| SE-Uncleaned < ME-AROMAagg | 4.782 | <0.001 | 4.782 | <0.001 |
| SE-Uncleaned < ME-ICA | 4.782 | <0.001 | 4.782 | <0.001 |
| MWC < FIXsoft | 2.335 | 0.02 | 0.771 | 0.441 |
| MWC < FIXagg | 4.782 | <0.001 | 4.782 | <0.001 |
| MWC < ICA-AROMAsoft | 4.782 | <0.001 | 4.782 | <0.001 |
| MWC < ICA-AROMAagg | 4.782 | <0.001 | 4.782 | <0.001 |
| MWC < ME-Uncleaned | 4.782 | <0.001 | 4.782 | <0.001 |
| MWC < ME-AROMAagg | 4.782 | <0.001 | 4.782 | <0.001 |
| MWC < ME-ICA | 4.782 | <0.001 | 4.782 | <0.001 |
| FIXsoft < FIXagg | 4.782 | <0.001 | 4.782 | <0.001 |
| FIXsoft < ICA-AROMAsoft | 3.898 | <0.001 | 4.247 | 0.001 |
| FIXsoft < ICA-AROMAagg | 4.350 | <0.001 | 4.741 | <0.001 |
| FIXsoft < ME-Uncleaned | 4.782 | 0.002 | 4.782 | <0.001 |
| FIXsoft < ME-AROMAagg | 4.782 | <0.001 | 4.782 | <0.001 |
| FIXsoft < ME-ICA | 4.782 | <0.001 | 4.782 | <0.001 |
| FIXagg < ICA-AROMAsoft | -2.540 | 0.011 | -2.458 | 0.014 |
| FIXagg < ICA-AROMAagg | 0.237 | 0.813 | 0.154 | 0.877 |
| FIXagg < ME-Uncleaned | 4.782 | <0.001 | 4.453 | 0.001 |
| FIXagg < ME-AROMAagg | 4.782 | <0.001 | 4.782 | <0.001 |
| FIXagg < ME-ICA | 4.782 | <0.001 | 4.782 | <0.001 |
| ICA-AROMAsoft < ICA-AROMAagg | 4.782 | <0.001 | 4.782 | <0.001 |
| ICA-AROMAsoft < ME-Uncleaned | 4.782 | <0.001 | 4.679 | <0.001 |
| ICA-AROMAsoft < ME-AROMAagg | 4.782 | <0.001 | 4.782 | <0.001 |
| ICA-AROMAsoft < ME-ICA | 4.782 | <0.001 | 4.782 | <0.001 |
| ICA-AROMAagg < ME-Uncleaned | 4.782 | <0.001 | 4.453 | 0.007 |
| ICA-AROMAagg < ME-AROMAagg | 4.782 | <0.001 | 4.782 | <0.001 |
| ICA-AROMAagg < ME-ICA | 4.782 | <0.001 | 4.782 | <0.001 |
| ME-Uncleaned < ME-AROMAagg | 4.782 | <0.001 | 4.782 | <0.001 |
| ME-Uncleaned < ME-ICA | 4.751 | <0.001 | 4.782 | <0.001 |
| ME-AROMAagg < ME-ICA | -4.782 | <0.001 | -4.762 | <0.001 |
