# Supplementary material for: Comparing resting state fMRI de-noising approaches using multi- and single-echo acquisitions
Source: PLoS One. 2017 Mar 21;12(3):e0173289. doi: 10.1371/journal.pone.0173289 (PMC5360253; doi:10.1371/journal.pone.0173289)
Supplement: S6 Table — For each comparison, the first and the second rows respectively report the results of HC and ADHD patients. (DOCX) [file pone.0173289.s018.docx]

**S6 Table. Comparison of FC between PCC and the other nodes of the DMN among different cleaning approaches.** For each comparison, the first and the second rows respectively report the results of HC and ADHD patients.

|  | **PCC-mPFC**  z-score  (p-value) | **PCC-left IPL**  z-score  (p-value) | **PCC-right IPL**  z-score  (p-value) | **PCC-left hippocampus**  z-score  (p-value) | **PCC-right hippocampus**  z-score  (p-value) |
| --- | --- | --- | --- | --- | --- |
| SE-Uncleaned < MWC | -2.952 (0.003) | -4.7  (<0.001) | -3.466 (0.001) | -3.425  (0.001) | -3.836  (<0.001) |
|  | -3.507 (<0.001) | -4.535 (<0.001) | -4.247  (<0.001) | -3.569  (<0.001) | -4.659  (<0.001) |
| SE-Uncleaned < FIXsoft | -1.306  (n.s) | -2.293  (0.022) | -2.232 (0.026) | -1.985  (0.047) | -2.725  (0.006) |
|  | -0.874  (n.s.) | -2.952  (0.003) | -3.342  (0.002) | -1.841  (n.s.) | -2.622  (0.009) |
| SE-Uncleaned < FIXagg | -2.561 (0.01) | -4.042  (<0.001) | -4.042 (<0.001) | -3.096  (0.002) | -3.383  (0.001) |
|  | -2.376 (0.018) | -4.227  (<0.001) | -4.268  (<0.001) | -2.849  (0.004) | -4.597  (<0.001) |
| SE-Uncleaned < ICA-AROMAsoft | 0.812  (n.s) | -0.175  (n.s.) | 1.944 (n.s.) | -0.422  (n.s.) | 0.051  (n.s.) |
|  | 1.368 (n.s.) | -1.573 (n.s.) | -0.977  (n.s.) | -0.771  (n.s.) | -1.512  (n.s.) |
| SE-Uncleaned < ICA-AROMAagg | -2.417 (0.016) | -3.569  (<0.001) | -2.499 (0.012) | -2.808  (0.005) | -2.787  (0.005) |
|  | -2.026  (0.043) | -4.35  (<0.001) | -4.33  (<0.001) | -3.26  (0.001) | -3.98  (<0.001) |
| SE-Uncleaned < ME-Uncleaned | 1.615  (n.s.) | -0.031  (n.s.) | 2.54  (0.011) | 1.573  (n.s.) | 3.404  (0.001) |
|  | 0.504 (n.s.) | -0.442 (n.s.) | 0.73  (n.s.) | 3.137  (0.002) | 3.013  (0.003) |
| SE-Uncleaned < ME-AROMAagg | 0.812 (n.s.) | -0.936  (n.s.) | 0.668 (n.s.) | -1.615  (n.s.) | -0.895  (n.s.) |
|  | 0.319 (n.s.) | -0.936  (n.s.) | -0.648  (n.s.) | -0.936  (n.s.) | -0.319  (n.s.) |
| SE-Uncleaned < ME-ICA | 3.959 (<0.001) | 2.417  (0.016) | 2.622  (0.009) | 4.062  (<0.001) | 4.247  (<0.001) |
|  | 3.383 (0.001) | 1.759  (n.s.) | 1.861  (n.s.) | 2.314  (0.021) | 3.857  (<0.001) |
| MWC < FIXsoft | 2.952  (0.003) | 3.034 (0.002) | 1.306 (n.s.) | 1.903  (n.s.) | 1.388  (n.s.) |
|  | 3.589 (<0.001) | 1.717  (n.s.) | -0.051  (n.s.) | 3.075  (0.002) | 2.849  (0.009) |
| MWC < FIXagg | 0.339  (n.s.) | -1.82  (n.s.) | -1.532 (n.s.) | -0.854  (n.s.) | -0.751  (0.453) |
|  | 2.149 (0.032) | -1.327  (n.s.) | -2.54  (0.011) | 0.751  (n.s.) | 0.977  (n.s.) |
| MWC < ICA-AROMAsoft | 2.540  (0.011) | 2.088  (0.037) | 3.219 (0.001) | 2.828  (0.005) | 3.198  (0.001) |
|  | 3.589 (<0.001) | 2.376  (0.018) | 3.013  (0.003) | 3.569  (<0.001) | 2.026  (0.043) |
| MWC < ICA-AROMAagg | 0.010  (n.s.) | -1.779  (n.s.) | 0.113 (n.s.) | -0.339  (n.s.) | 0.298  (n.s.) |
|  | 1.080 (n.s.) | -1.697  (n.s.) | -2.499  (0.012) | 0.051  (n.s.) | -0.093  (n.s.) |
| MWC < ME-Uncleaned | 3.527  (<0.001) | 3.733 (<0.001) | 4.391 (<0.001) | 3.219  (0.001) | 4.35  (<0.001) |
|  | 2.828 (0.005) | 3.507 (<0.001) | 3.939 (<0.001) | 4.145 (<0.001) | 4.412  (<0.001) |
| MWC < ME-AROMAagg | 2.89  (0.004) | 1.676  (n.s.) | 2.766 (0.006) | 1.779  (n.s.) | 2.005  (0.045) |
|  | 2.458 (0.014) | 2.458 (0.014) | 2.684  (0.007) | 2.478  (0.013) | 3.425  (0.001) |
| MWC < ME-ICA | 4.618  (<0.001) | 4.535  (<0.001) | 3.815 (<0.001) | 4.659  (<0.001) | 4.638  (<0.001) |
|  | 4.247 (<0.001) | 3.836 (<0.001) | 3.774  (<0.001) | 4.083  (<0.001) | 4.453  (<0.001) |
| FIXsoft < FIXagg | -2.232 (0.026) | -4.247 (<0.001) | -2.931 (0.003) | -2.828  (0.005) | -2.622  (0.009) |
|  | -1.923 (n.s.) | -2.746 (0.006) | -2.89  (0.004) | -3.075  (0.002) | -2.376  (0.018) |
| FIXsoft < ICA-AROMAsoft | 1.656  (n.s.) | 1.183  (n.s.) | 3.013 (0.003) | 1.553  (n.s.) | 2.808  (0.005) |
|  | 2.602 (0.009) | 1.491 (n.s.) | 2.417 (0.016) | 1.944  (n.s.) | 0.422  (n.s.) |
| FIXsoft < ICA-AROMAagg | -1.8  (n.s.) | -3.075 (0.002) | -0.319  (n.s.) | -1.985  (0.047) | -0.956  (n.s.) |
|  | -1.306 (n.s.) | -2.396 (0.017) | -2.52  (0.012) | -3.034  (0.002) | -2.458  (0.014) |
| FIXsoft < ME-Uncleaned | 2.211 (0.027) | 1.861 (n.s.) | 3.692 (<0.001) | 2.396  (0.017) | 3.527  (<0.001) |
|  | 1.286 (n.s.) | 2.129 (0.033) | 3.815  (<0.001) | 2.931  (0.003) | 3.589  (<0.001) |
| FIXsoft < ME-AROMAagg | 1.512 (n.s.) | 0.36  (n.s.) | 2.417 (0.016) | 0.668  (n.s.) | 1.08  (n.s.) |
|  | 1.1  (n.s.) | 1.656 (n.s.) | 2.314  (0.021) | 0.895  (n.s.) | 2.108  (0.035) |
| FIXsoft < ME-ICA | 4.227 (<0.001) | 3.877 (<0.001) | 3.713 (<0.001) | 4.597  (<0.001) | 4.33  (<0.001) |
|  | 3.589 (<0.001) | 3.178 (0.001) | 3.713 (<0.001) | 3.157  (0.002) | 4.227  (<0.001) |
| FIXagg < ICA-AROMAsoft | 2.478  (0.013) | 2.931 (0.003) | 3.651 (<0.001) | 3.425  (0.001) | 3.959  (<0.001) |
|  | 3.116 (0.002) | 2.622 (0.009) | 3.26  (0.001) | 3.466  (0.002) | 2.047  (0.041) |
| FIXagg < ICA-AROMAagg | -0.442  (n.s.) | -0.998 (n.s.) | 1.82  (n.s.) | 0.113  (n.s.) | 1.121  (n.s.) |
|  | -0.442 (n.s.) | -0.915  (n.s.) | -0.113  (n.s.) | -1.183  (n.s.) | -1.224  (n.s.) |
| FIXagg < ME-Uncleaned | 3.034 (0.002) | 3.774  (<0.001) | 4.535 (<0.001) | 3.301  (0.001) | 4.124  (<0.001) |
|  | 2.067 (0.039) | 3.445 (0.001) | 4.453 (<0.001) | 3.322  (0.001) | 4.206  (<0.001) |
| FIXagg < ME-AROMAagg | 2.664  (0.008) | 2.335 (0.02) | 3.507 (<0.001) | 2.828  (0.005) | 2.993  (0.003) |
|  | 1.409 (n.s.) | 2.622 (0.009) | 2.952 (0.003) | 2.293  (0.022) | 3.507  (<0.001) |
| FIXagg < ME-ICA | 4.412  (<0.001) | 4.576  (<0.001) | 4.021 (<0.001) | 4.679  (<0.001) | 4.638  (<0.001) |
|  | 4.042 (<0.001) | 3.774 (<0.001) | 4.165 (<0.001) | 3.918  (<0.001) | 4.371  (<0.001) |
| ICA-AROMAsoft < ICA-AROMAagg | -4.33  (<0.001) | -.412 (<0.001) | -4.432 (<0.001) | -4.186  (<0.001) | -4.33  (<0.001) |
|  | -4.741 (<0.001) | -4.659 (<0.001) | -4.618 (<0.001) | -4.782  (<0.001) | -3.836  (<0.001) |
| ICA-AROMAsoft < ME-Uncleaned | 0.381  (n.s.) | 0.71  (n.s.) | 0.36  (n.s.) | 0.854  (n.s.) | 1.985  (0.047) |
|  | -0.854 (n.s.) | 1.306 (n.s.) | 1.882  (n.s.) | 2.026  (0.043) | 3.486  (<0.001) |
| ICA-AROMAsoft < ME-AROMAagg | 0.093  (n.s.) | -0.463  (n.s.) | -0.648  (n.s.) | -0.977  (n.s.) | -1.08  (n.s.) |
|  | -0.792  (n.s.) | 0.216  (n.s.) | 0.73  (n.s.) | -0.216  (n.s.) | 1.656  (n.s.) |
| ICA-AROMAsoft < ME-ICA | 3.013  (0.003) | 2.355  (0.019) | 1.183  (n.s.) | 3.939  (<0.001) | 3.695  (<0.001) |
|  | 1.759 (n.s.) | 2.17 (0.03) | 2.335 (0.02) | 2.705  (0.007) | 4.186  (<0.001) |
| ICA-AROMAagg < ME-Uncleaned | 3.157  (0.002) | 3.363 (0.001) | 3.733  (<0.001) | 2.952  (0.003) | 3.713  (<0.001) |
|  | 1.882 (n.s.) | 4.042  (<0.001) | 4.288 (<0.001) | 3.877  (<0.001) | 4.206  (<0.001) |
| ICA-AROMAagg < ME-AROMAagg | 2.931  (0.003) | 3.404 (0.001) | 2.808 (0.005) | 2.520  (0.012) | 2.52  (0.012) |
|  | 1.964 (0.049) | 3.445  (0.002) | 3.959 (<0.001) | 3.24  (0.001) | 4.206  (<0.001) |
| ICA-AROMAagg < ME-ICA | 4.309  (<0.001) | 4.206 (<0.001) | 3.754  (<0.001) | 4.618  (<0.001) | 4.515  (<0.001) |
|  | 3.692 (<0.001) | 4.124 (<0.001) | 4.042 (<0.001) | 4.247  (<0.001) | 4.371  (<0.001) |
| ME-Uncleaned < ME-AROMAagg | -0.668  (n.s.) | -0.915 (n.s.) | -1.697  (n.s.) | -2.108  (0.035) | -2.849  (0.004) |
|  | -0.031 (n.s.) | -0.36  (n.s.) | -1.409  (n.s.) | -2.376  (0.018) | -2.643  (0.008) |
| ME-Uncleaned < ME-ICA | 4.103  (<0.001) | 3.754 (<0.001) | 1.882 (n.s.) | 3.774  (<0.001) | 3.774  (<0.001) |
|  | 3.836 (<0.001) | 2.643 (0.008) | 1.656  (n.s.) | 1.388  (n.s.) | 2.314  (0.021) |
| ME-AROMAagg < ME-ICA | 3.013  (<0.001) | 3.342  (0.001) | 2.622 (0.009) | 4.021  (<0.001) | 4.268  (<0.001) |
|  | 2.828 (0.005) | 1.82  (n.s.) | 2.149 (0.032) | 3.281  (0.001) | 3.486  (<0.001) |
