# Supplementary figures and images for: Comparing resting state fMRI de-noising approaches using multi- and single-echo acquisitions
Source: PLoS One. 2017 Mar 21;12(3):e0173289. doi: 10.1371/journal.pone.0173289 (PMC5360253; doi:10.1371/journal.pone.0173289)

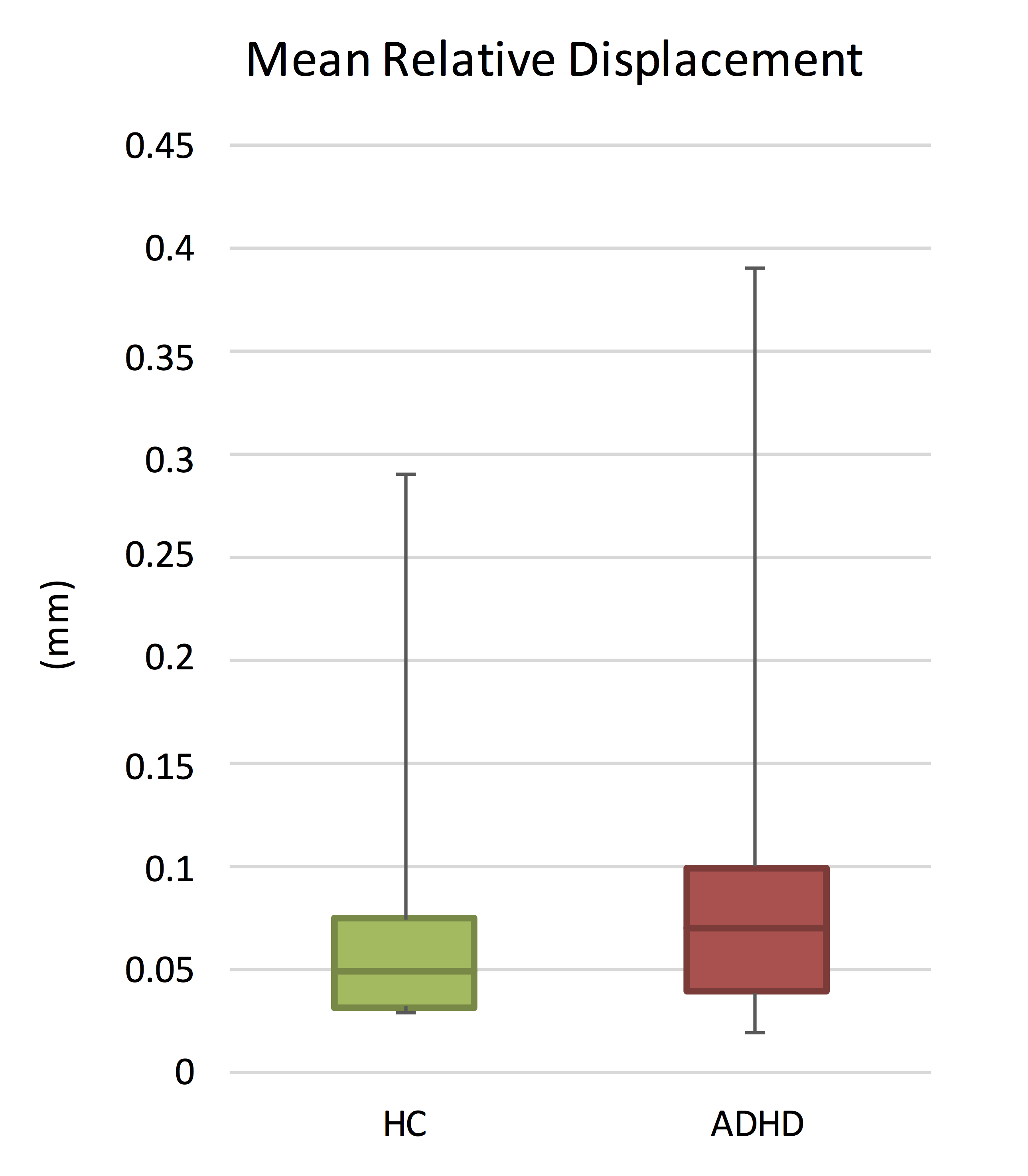

Supplement: S1 Fig — (TIFF) [file pone.0173289.s019.tiff]

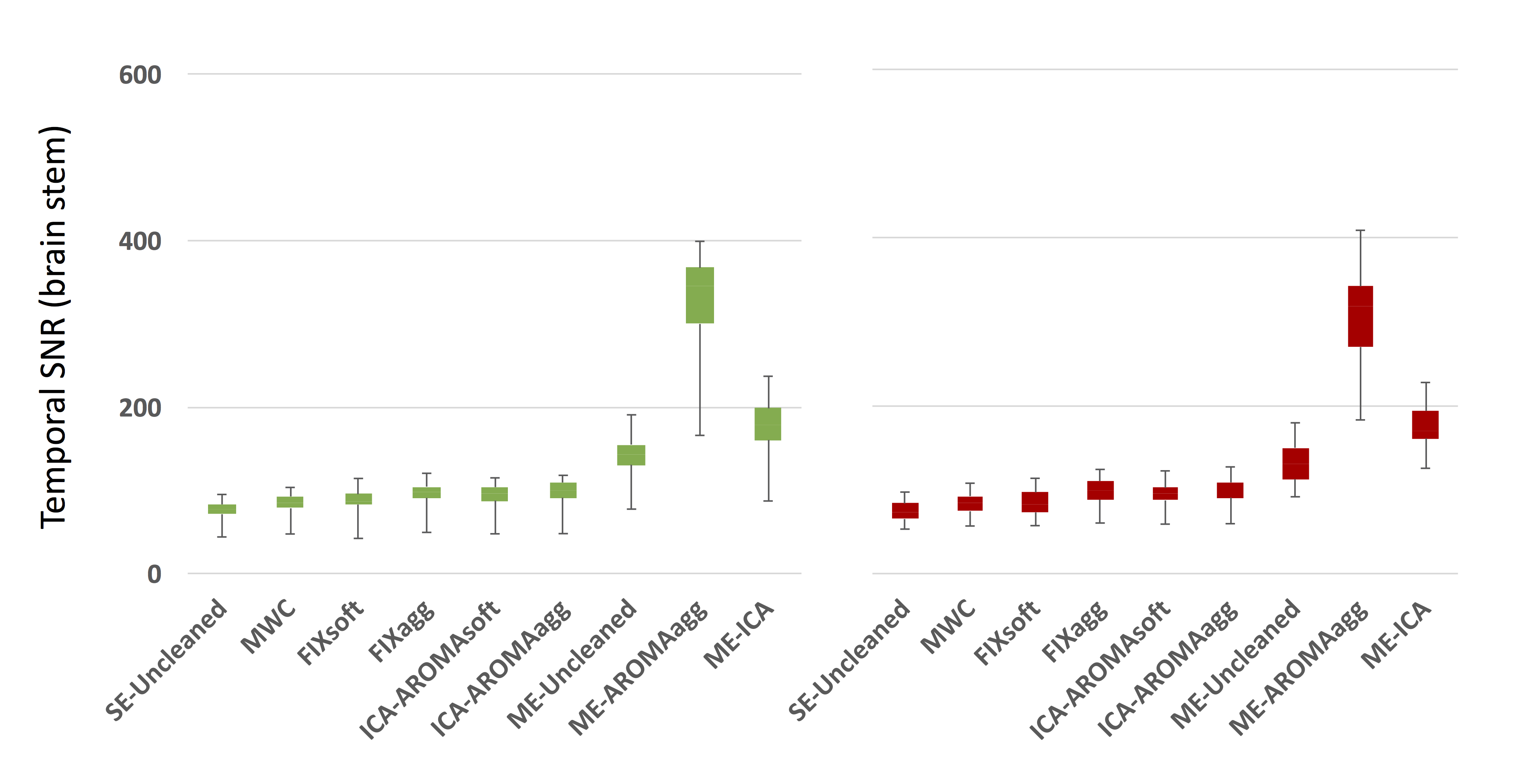

Supplement: S2 Fig — (TIFF) [file pone.0173289.s020.tiff]

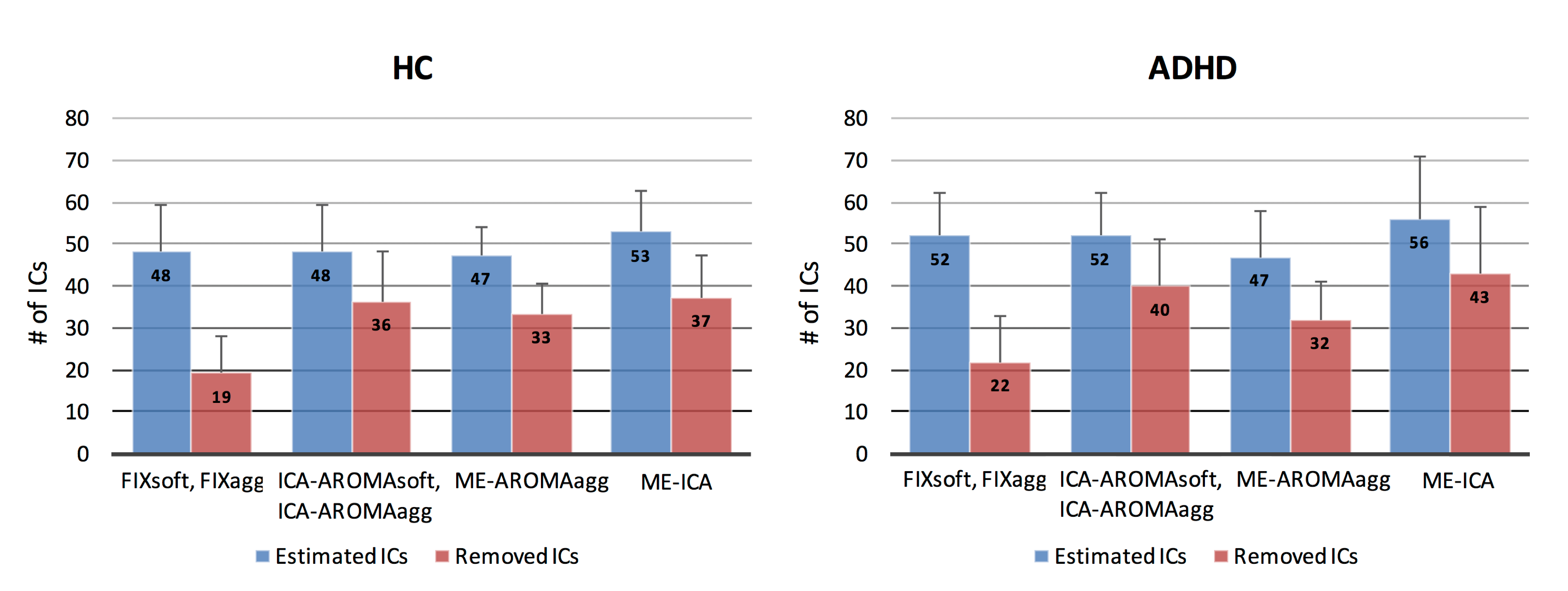

Supplement: S3 Fig — Numbers within the bars indicate the average number of estimated and removed components for each method and group. The whiskers indicate the standard deviation. (TIFF) [file pone.0173289.s021.tiff]

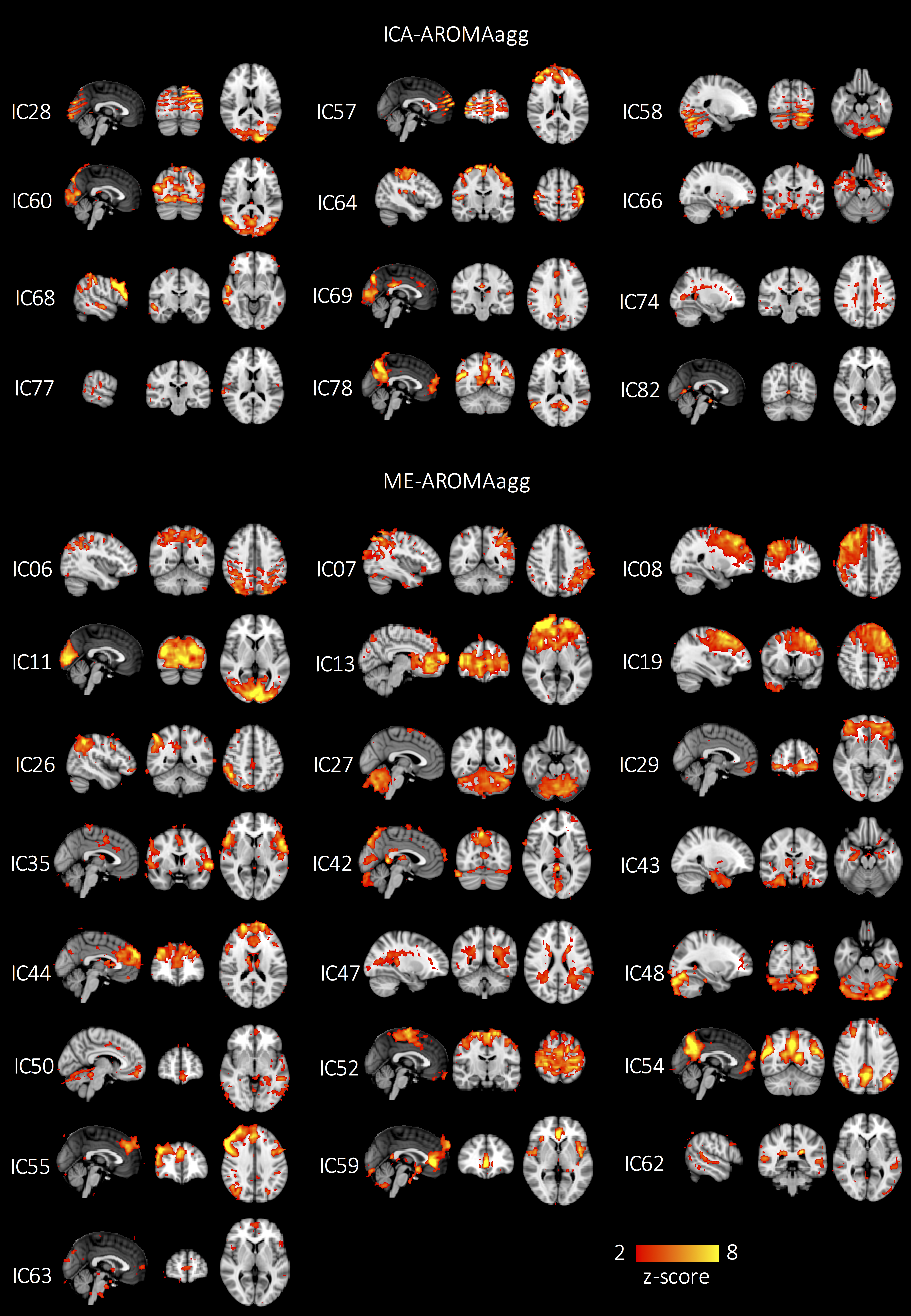

Supplement: S4 Fig — For this specific subject, ICA-AROMAagg decomposed the signal into 83 ICs and recognized as good only 12 of them (upper panel). Among the good ICs, stripe artifacts are clearly seen in the first three components (IC28, IC57 and IC58), which can still be recognized as resting state networks as include areas respectively belonging to the visual network, executive network and the cerebellum. ME-AROMAagg recognized 22 good ICs out of 66, which is more than twice the good ICA-AROMAagg ICs. However, even if ME-AROMAagg preserved more variance contribution than ICA-AROMAagg, some components (e.g., IC8, IC19, IC42) still presented residual noise. (TIFF) [file pone.0173289.s022.tiff]

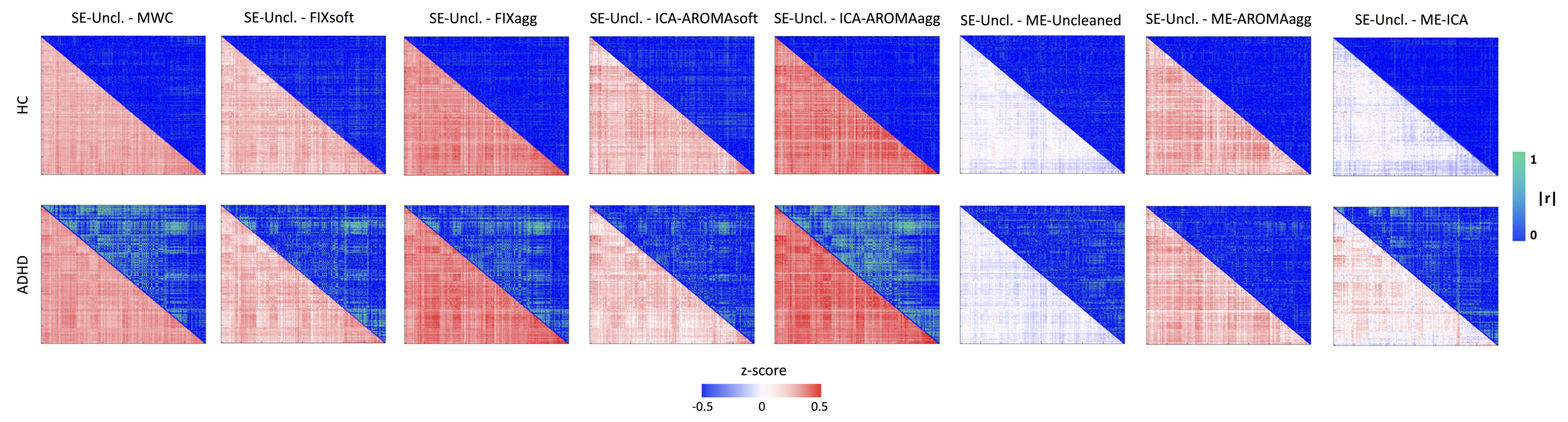

Supplement: S5 Fig — Each lower triangular matrix represents the group-averaged difference between the SE-Uncleaned FC matrix and the de-noised one (HC in the first row, ADHD patients in the second one) for each cleaning procedure. The upper triangular matrices show the correlation across subjects between the spurious FC and the mean relative displacement. (TIFF) [file pone.0173289.s023.tiff]

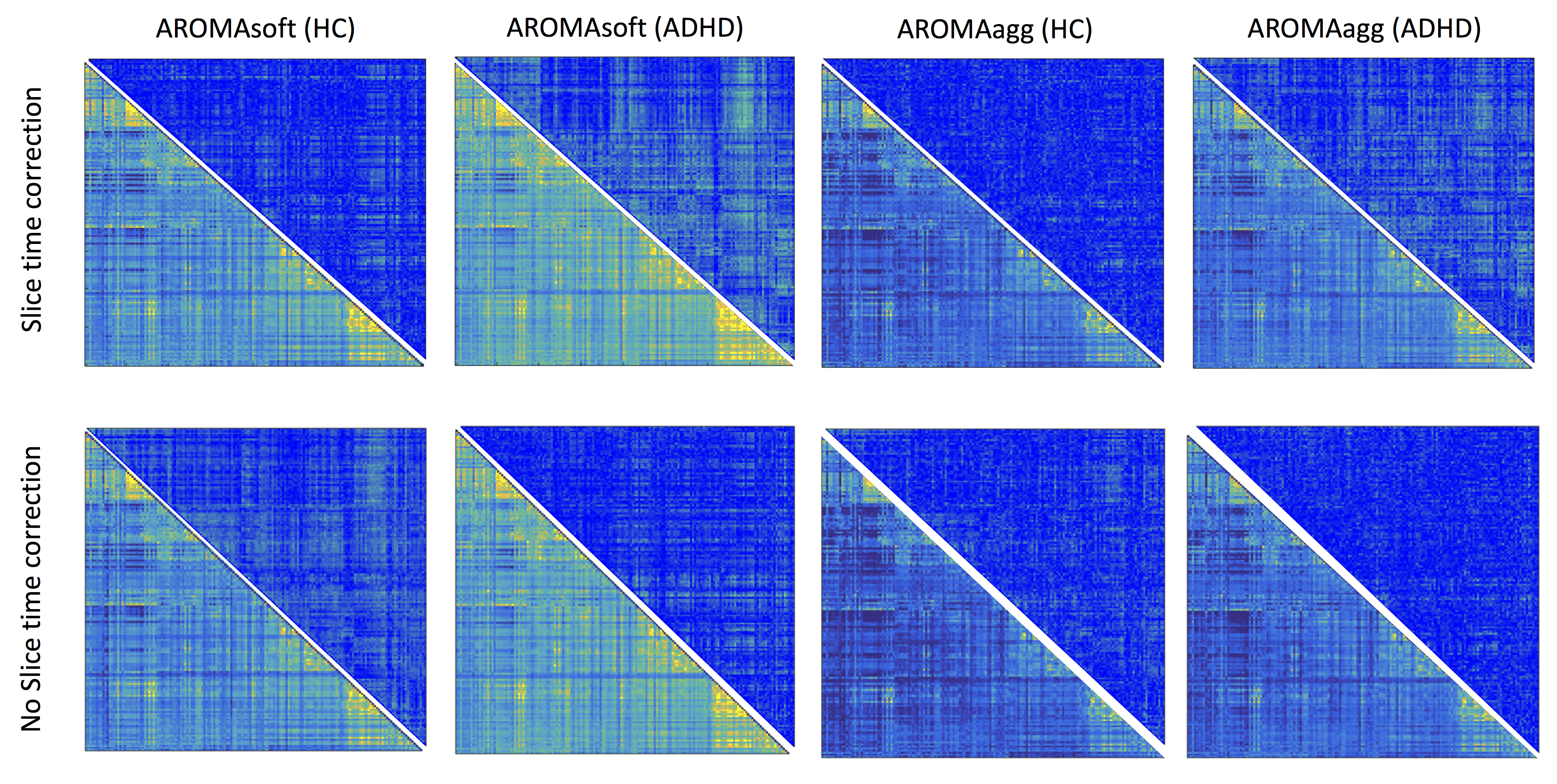

Supplement: S6 Fig — (TIFF) [file pone.0173289.s024.tiff]

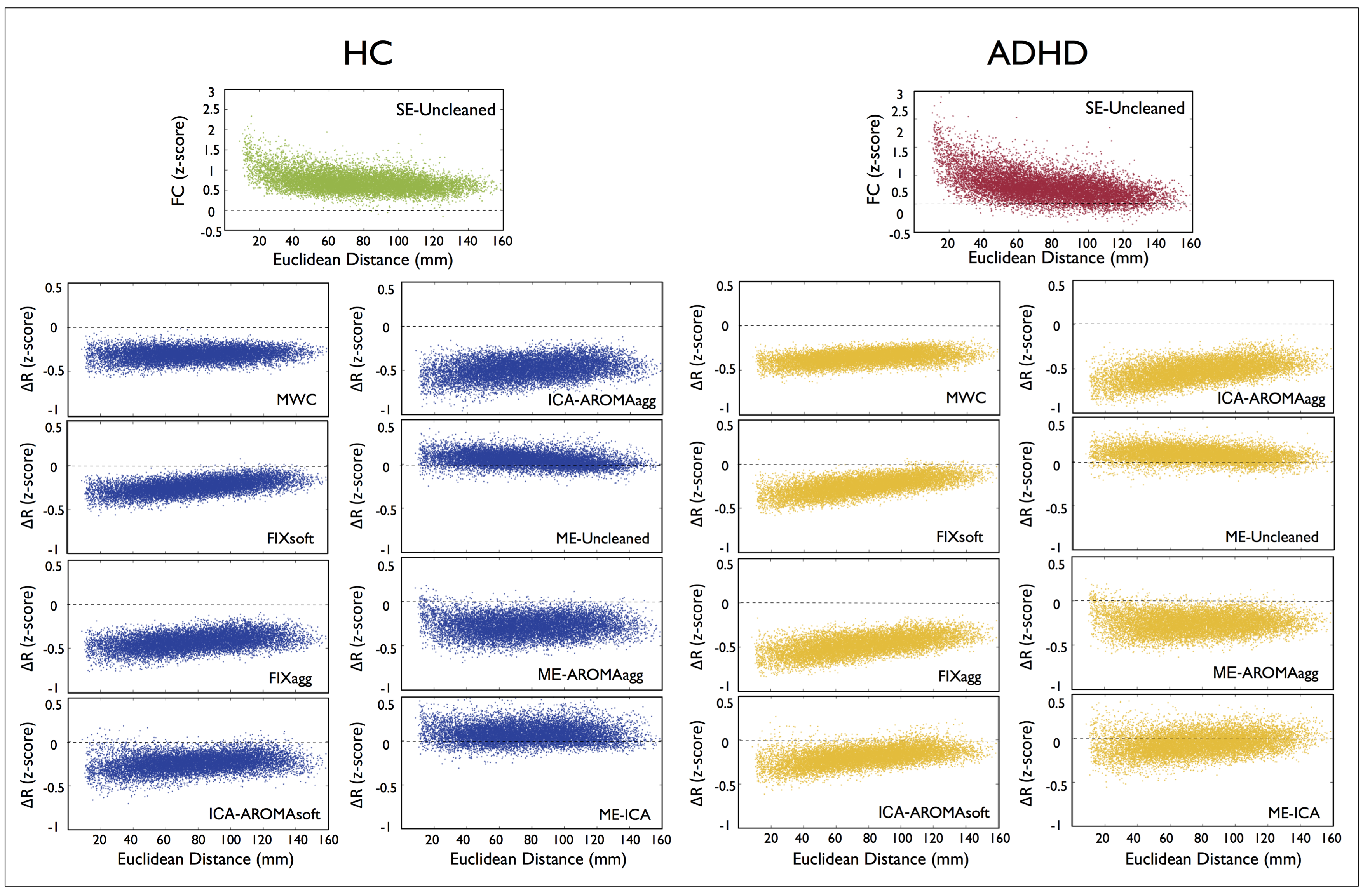

Supplement: S7 Fig — The two scatter plots at the top show the dependency of ROI-to-ROI FC on the Euclidean distance between nodes in the SE-Uncleaned data of both groups (green dots for the HC group; red dots for the ADHD group). The ΔR scatter plots on the bottom (blue dots for the HC group; yellow dots for the ADHD group) represent the distribution of the difference in FC between de-noised data and SE-Uncleaned data as a function of the Euclidean distance between the nodes. Single-echo methods and ME-AROMAagg reduced the FC between all nodes in both groups independent of the anatomical distance between them. ME-ICA reduced the short-range functional connections and increased long-range connections in the ADHD group. (TIFF) [file pone.0173289.s025.tiff]

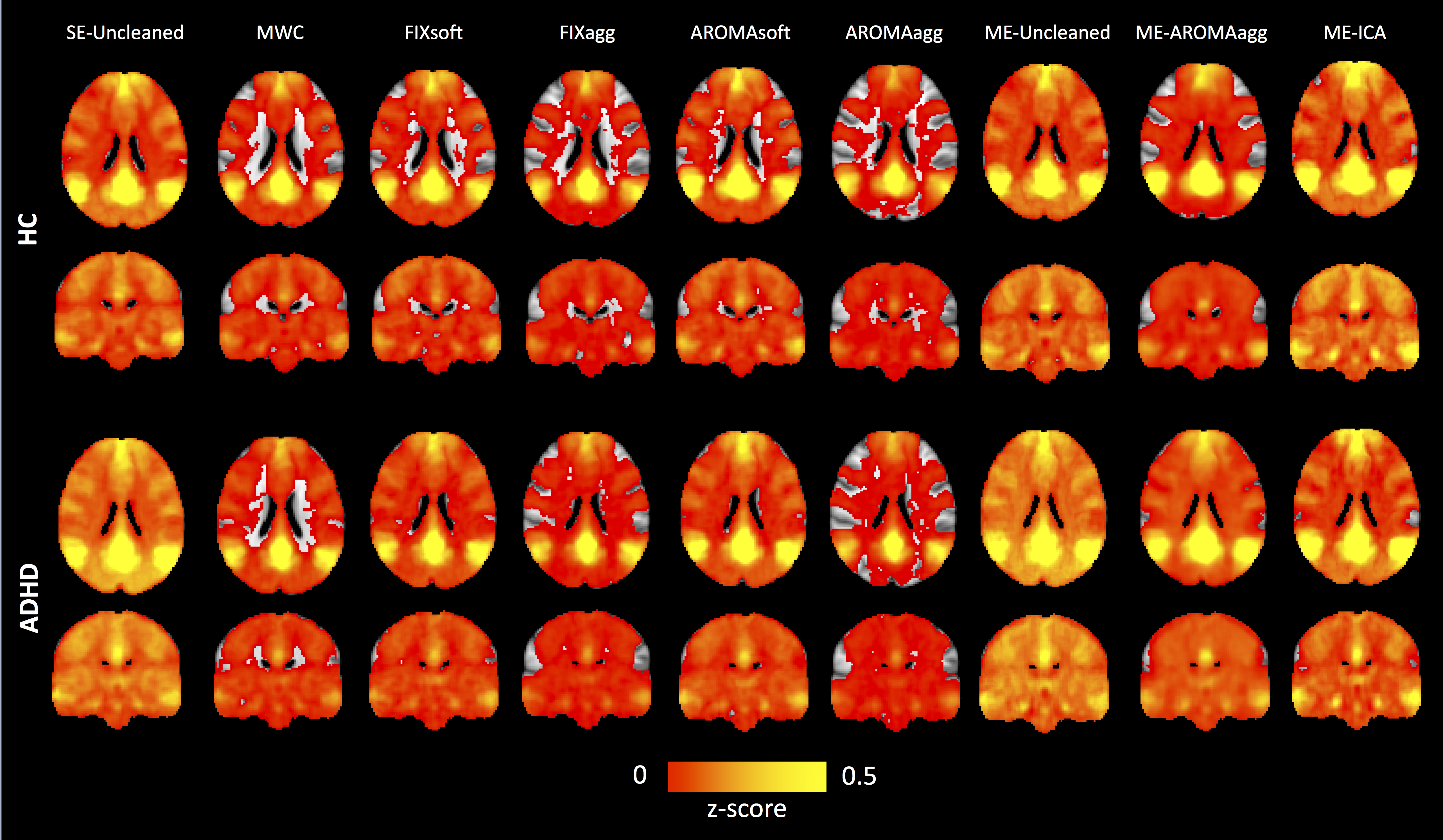

Supplement: S8 Fig — These maps are the same of those reported in Fig 7, with the only difference that an arbitrary threshold of the z-score was not applied here. (TIFF) [file pone.0173289.s026.tiff]

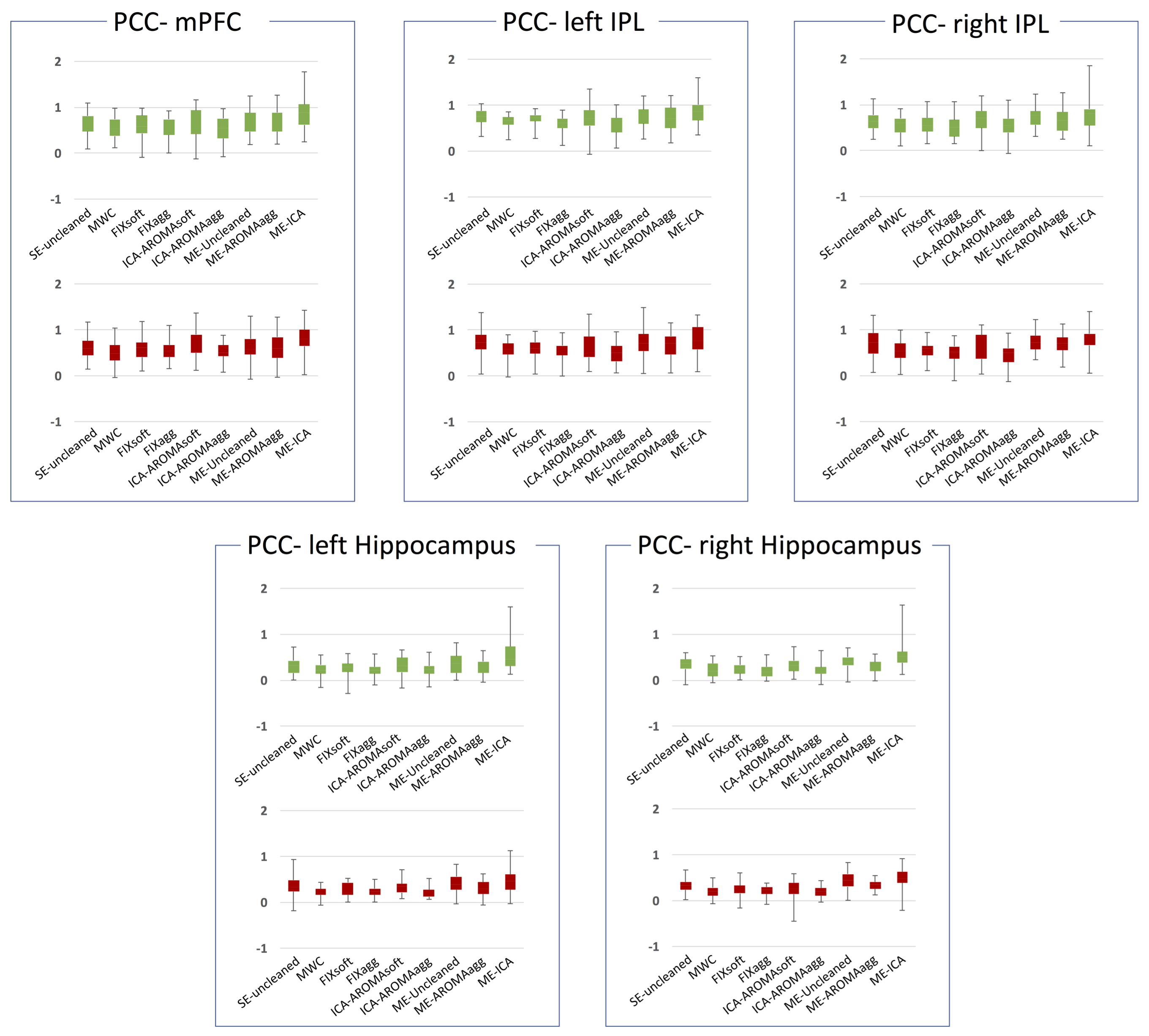

Supplement: S9 Fig — (TIFF) [file pone.0173289.s027.tiff]
